# The engagement of the cerebellum and basal ganglia enhances expertise in a sensorimotor adaptation task

**DOI:** 10.1162/imag_a_00271

**Published:** 2024-08-19

**Authors:** Joshua B. Tan, Eli Müller, Andrii Zahorodnii, James M. Shine

**Affiliations:** Brain and Mind Centre, School of Medical Sciences, Faculty of Medicine and Health, University of Sydney, Sydney, Australia; Centre for Complex Systems, School of Physics, University of Sydney, Sydney, Australia; Massachusetts Institute of Technology, Boston, MA, United States

**Keywords:** adaptation, cerebellum, basal ganglia, sensorimotor, fMRI

## Abstract

The ability to adapt to changes in the environment is essential for skilled performance, especially in competitive sports and events, where experts consistently perform at the highest level, rapidly adapting to unpredictable conditions. Current studies have identified cortical-cortical interactions between the premotor and primary motor cortex during expert performance; however, while these interactions are important for planning and execution, our understanding of the mechanisms underlying learning, feedback, and adaptation remains unclear. Subcortical structures, such as the cerebellum, have dense connections with the cerebral cortex through which they provide precise topological constraints that could putatively play a crucial role in fast, accurate task execution. To test this hypothesis, we tracked cortical, subcortical, and cerebellar BOLD activity during a visuomotor rotation task in which participants executed a visual cue-driven, ballistic motor task across three conditions: at baseline; following a 45° clockwise motor rotational perturbation; and then within a follow-up (washout) condition. We observed increased recruitment of primary visual, basal ganglia, and cerebellar regions that robustly covaried with fast, accurate performance across all conditions (baseline, rotation, and washout). Tracking individualised performance across participants, we observed three distinct groups: experts (consistently fast and accurate), adapters (initially poor with improvement to expert-level), and non-adapters (initially good but ultimately poor performance). The experts and adapter groups demonstrated performances that were robust to changes in conditions and were more variable in their neural signatures between trials, whereas the performance of non-adapters decreased with changes in conditions and were characterised by less variable neural signatures. These results aligned with the tenets of the differential learning theory. To establish the validity of our interpretation of these whole-brain signatures and behavioural patterns, the neuroimaging results were reproduced by training recurrent neural networks representing each group and analysing their resultant activity patterns. Together, these results provide evidence for cerebellar and basal ganglia contributions to expertise in adaptation and suggest a possible connection between variable brain patterns and robust performance.

## 1 Introduction

A defining characteristic of expertise is the ability to adapt—to perform at high levels across multiple scenarios, including surprising and novel situations (Bransford & Schwartz, 1999;Cheung & Kulasegaram, 2022). At high levels of sports (e.g., tennis), adaptation is crucial. For instance, an athlete (such as a tennis player) has to adjust to perturbations caused by environmental factors (e.g., effects of wind and temperature on ball trajectories) or from the opposing player (e.g., different types of spins on the ball). Similarly, adaptation is important in cognitive tasks, including tasks that require translating skills from one situation to another (e.g., playing a game of chess after losing a Queen). However, between individuals, there can be differences in how fast one adapts to changes as well as what strategies are used to adapt (McDougle et al., 2016;Schollhorn et al., 2012;Taylor & Ivry, 2011). Hence adaptation is a complex skill applicable in both physical and mental activities, and understanding how to optimally adapt would lead to improved performance and expertise.

Understanding the neural correlates of actions both physical and cognitive is inherently difficult. Within a single action, multiple processes are undertaken—both sequentially and in parallel—however, to isolate specific sub-components of actions, experimental tasks are often designed in order to isolate a specific event. Furthermore, with limitations in signal strength during standard neural recordings, experimental tasks can range from being so simple that the specific function that is isolated bears no resemblance of the desired action; to so complex that the new task requires the execution of an entirely new movement. Motor adaptation tasks offer a work-around, in that they accurately simplify natural behaviours through tasks that anyone can perform, while still modifying a participant’s performance by altering specific task rules without changing the difficulty of the task (Krakauer, 2009;Tsay et al., 2022). There are numerous versions of motor adaptation tasks that evoke different sensory adaptations, such as visuomotor rotations (Areshenkoff et al., 2022;Krakauer, 2009;Perich et al., 2018) and force-field perturbations (Perich & Miller, 2017;Perich et al., 2018). Importantly, these tasks have been performed during simultaneous functional neuroimaging (Areshenkoff et al., 2022;Perich & Miller, 2017;Perich et al., 2018), and can also be decoded using neural networks (Pemberton et al., 2022). By consolidating findings across modalities and techniques, an understanding of how the brain adapts to changes in the environment can be attained. Thus begs the question, how might the brain reconfigure its activity to mediate effective adaptation?

In the past decade, both technological and methodological advances have improved our understanding of adaptation in the brain; however, a comprehensive understanding of how the brain as a whole learns to adapt to perturbations remains unclear. The current motor learning literature has a strong focus towards the cerebral cortex and cortical-cortical interactions, with the consensus approach highlighting how primary motor regions change with learning and with different inputs (Churchland et al., 2012,^2006^;Gallego et al., 2020;Perich et al., 2018). As the relationship between primary motor regions and movement dynamics remains unchanged during adaptive behaviour (Perich & Miller, 2017), connections between primary motor regions and other cerebral cortical regions (such as the premotor cortex) may serve an important role in adaptive learning. However, these cortical-cortical interactions are unable to capture the complete scope of motor learning, such as how the brain as a whole processes error feedback (Cohen et al., 2017;Prut et al., 2001) and the decrease in cortical regions over the duration of a task (Bassett et al., 2015;Berlot et al., 2020). Furthermore, premotor and motor regions share connections with numerous other subcortical structures of the brain such as the basal ganglia, thalamus, and cerebellum (Kelly & Strick, 2003;Middleton & Strick, 1994;Shine, 2021). Perhaps most damning for cortico-centric hypotheses is that much of the motor cortex can be surgically removed in expert mice, without an observable decline in performance (Kawai et al., 2015;Wolff et al., 2022). Hence, a cerebral cortico-centric focus on motor learning and adaptation does not justify the complexity of the anatomical connections within the brain nor explain the wide variations in behaviour.

Although the basal ganglia has long been associated with the execution of habitual behaviours (Graybiel & Grafton, 2015;Yin & Knowlton, 2006), there are other subcortical structures—such as the cerebellum (Gilbert & Thach, 1977;Rotondo et al., 2022)—that are well-suited to facilitate adaptive behaviour, but have not received sufficient theoretical attention. Consisting of more than 50% of the total neurons in the whole brain (Wagner et al., 2019;Zagon et al., 1977), the cerebellum has a repeated, modular structure that integrates the cerebellar cortex with the cerebral cortex, both via inputs (via the cortico-pontine-cerebellar system) and outputs (via the cerebello-thalamo-cortical circuits) (Albus, 1971;Koziol et al., 2014;Nashef et al., 2018), as well as with the spinal cord (Albus, 1971;Cohen et al., 2017;Zinger et al., 2013). The modular structure of the cerebellum allows for compartmentalisation of unique inputs (Hayter et al., 2007;Kostadinov & Häusser, 2022), the learning and execution of complex, nested sequences of input-output patterns (Khilkevich et al., 2018), as well as more complex, multi-domain functions like parallel processing (Müller et al., 2023). Furthermore, the cerebellum has also been linked to pattern separation and feed-forward predictions, akin to a forward model for effective motor control (Bastian, 2006;Imamizu & Kawato, 2009;Ramnani, 2014;Stein, 2021;Wolpert et al., 1998). These functions play pivotal roles in adaptation and learning, as pattern separation allows the brain to disentangle similar actions from each other, and feed-forward mechanisms facilitate rapid learning; however, to date, few studies have investigated this systems-level perspective in adaptation tasks. Feed-forward mechanisms or the anticipation of movement outcomes have previously been linked with learning an internal model of the task—where the internal model refers to encoding the task in the brain (e.g., understanding how a tennis racquet would move when your arm moves) (Imamizu et al., 2000;Wolpert et al., 1998). In adaptation paradigms, the cerebellum has been suggested to serve a role in developing and storing internal models through implicit learning and error feedback (Doyon & Benali, 2005;Doyon et al., 2003;Hikosaka et al., 2002;Imamizu et al., 2000;Mazzoni & Krakauer, 2006;Tseng et al., 2007). Importantly, both lesioning of the cerebellum (Sendhilnathan et al., 2024) and patients with cerebellar ataxia (Morton & Bastian, 2006) resulted in impaired adaptation specifically learning in-between trials (Tzvi et al., 2022). Hence, the cerebellum facilitates learning in adaptation by contributing important functions such as pattern separation and feed-forward predictions.

Another key subcortical structure involved in adaptation and learning are the basal ganglia (Foerde & Shohamy, 2011;Lanciego et al., 2012) which are a small group of nuclei with internal inhibitory connectivity and a modulatory (GABAergic) role controlling thalamic firing (Nambu, 2008;Wilson, 2013). The basal ganglia are also disynaptically connected with the cerebellum (Bostan & Strick, 2018;Todorov et al., 2019), though the precise interplay between these two structures remains somewhat enigmatic. Therefore, approaching expertise and adaptation from a systems-level perspective that involves interactions between the cerebellum, basal ganglia, and cortical regions would enable better adjustments to perturbations and increase the speed of learning.

Here, we analyse BOLD fMRI patterns within the cerebral cortex, cerebellum, and basal ganglia during a sensorimotor adaptation task to determine the relationship between these structures following a perturbation to the sensorimotor contingencies inherent within the task (i.e., a 45° rotation to cursor movements). We hypothesised that increased performance in the adaptation task would be associated with an increased response within the cerebellum and basal ganglia. To tackle the inter-individual differences during learning, we compared differences between trials using a data-driven clustering approach. Based on the differential learning theory (Frank et al., 2008), we predicted that variation in strategies during learning would drive inter-individual differences, with individuals who had more variation during learning adapting faster to perturbations. Finally, we created a simple recurrent neural network model to test the validity of this predicted mechanism. Overall, our results provide important evidence to support distributed cortico-cerebellar circuits in sensorimotor adaptation.

## 2 Methods

### 2.1 Participant demographics

Forty right-handed individuals between the ages of 18 and 35 (mean = 22.5, SD = 4.51; 13 males) participated in the study and received financial compensation for their time. Data from eight participants were excluded due to either head motion in the MRI scanner (N = 4; motion greater than 2 mm or 2° rotation within a single scan) or their inability to learn the rotation task (N = 4). The final dataset consisted of 32 participants. Right-handedness was assessed with the Edinburgh handedness questionnaire (Oldfield, 1971). Participants’ written, informed consent was obtained before commencement of the experimental protocol. The Queen’s University Research Ethics Board approved the study, and it was conducted in accordance with the principles outlined in the Canadian Tri-Council Policy Statement on Ethical Conduct for Research Involving Humans and the principles of the Declaration of Helsinki (World Medical Association, 2013). Data were obtained from a previous study (Standage et al., 2023) and are available on the*OpenNeuro*repository (accession number ds004021).

### 2.2 Study design

Participants performed hand movements directed toward a target by applying a directional force onto an MRI-compatible force sensor (ATI technologies) using their right index finger and thumb. Once directional force was applied, there was no ability to adjust directions until the next trial began. The target and cursor stimuli were rear-projected with an LCD projector (NEC LT265 DLP projector, 1,024 × 768 resolution, 60 Hz refresh rate) onto a screen mounted behind the participant. The stimuli on the screen were viewed through a mirror fixed to the MRI coil directly above participants’ eyes, thus preventing participants from being able to see their hand. The force sensor and associated cursor movement were sampled at 500 Hz.

**Fig. 1.**
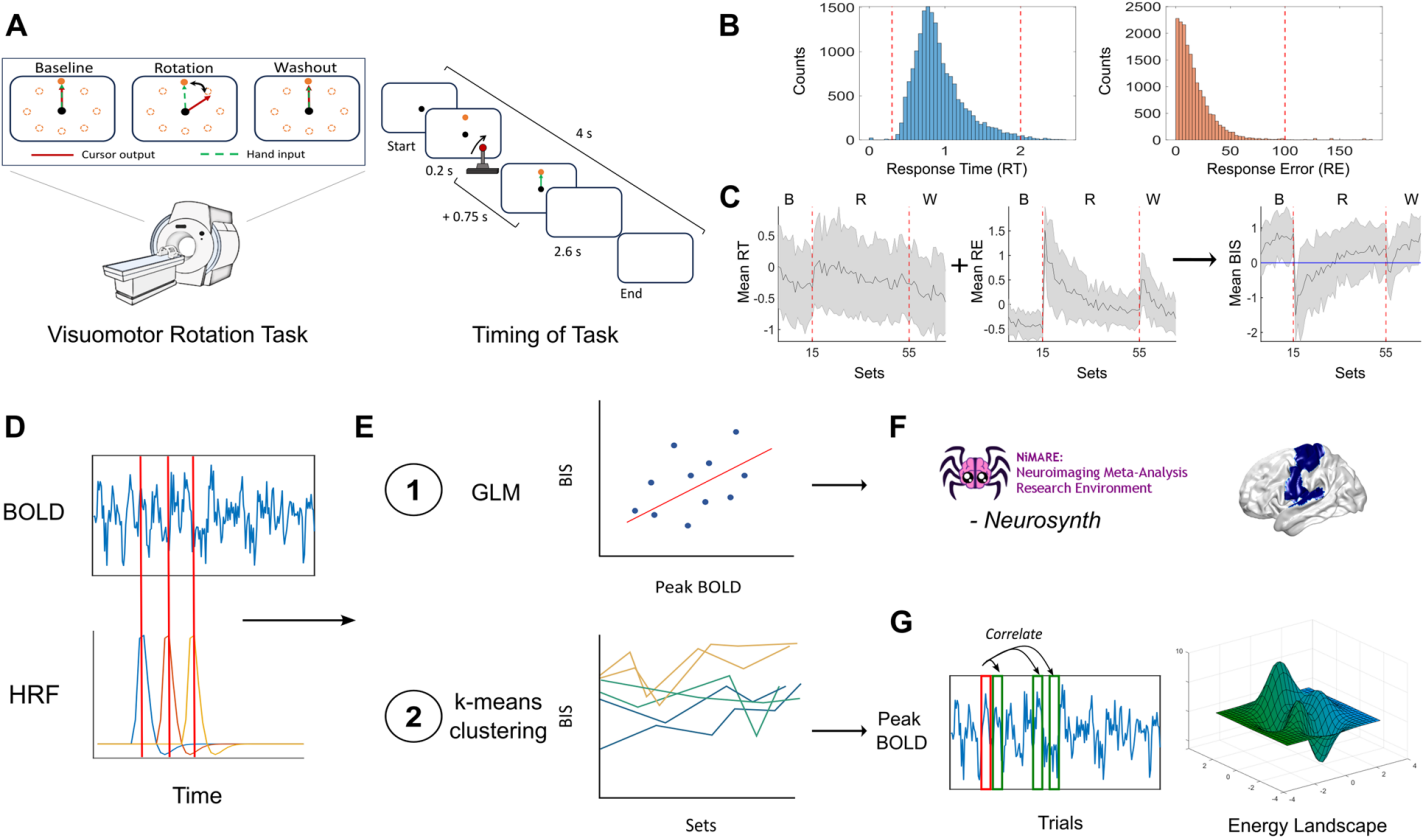
Summary of methods. (A) Task design including the three conditions, timing of stimuli, and response. (B) Thresholding of behaviour measurements. (C) Calculation of balanced integration score (BIS), and selection of individuals that completed the task competently. (D) Matching the trial onset time convolved by the haemodynamic response function (HRF) with the BOLD timeseries (peak BOLD). (E) General linear model (GLM) fitting BIS to peak BOLD timeseries for each participant. K-means clustering was applied using the BIS scores. (F) Comparing the generated brain map from the GLM with topics from the neurosynth database. (G) Analysing variance and inter-trial changes in brain responses using correlation and energy landscape analyses.

This experiment used a well-established VMR task (Krakauer, 2009) to probe sensorimotor adaptation. To start each trial, the cursor (20-pixel radius) appeared in a central start position (25-pixel radius). A white target circle (30-pixel radius) appeared at one of eight locations (0°, 45°, 90°, 135°, 180°, 225°, 270°, 315°) on an invisible ring around the central position (300-pixel radius) and filled in (white) following a 200 ms delay. Participants then applied a directional force on the sensor resulting in the cursor being launched towards the input direction. Once launched, the cursor would travel the 300-pixel distance to the invisible ring over a 750 ms period (with a bell-shaped velocity profile) before becoming stationary at the ring to provide participants with end-point error feedback. End-point error feedback is provided by the target white circle changing colours. If the cursor overlapped with the target to any extent, the target would become green, signifying a target “hit,” and if there was no overlap the target would remain white. Each trial was separated by 4 s and within this period, participants had 2.6 s from target presentation to complete the trial (including the 200 ms target delay, participants’ own reaction time, and the 750 ms cursor movement; any remaining time was allotted to providing the end-point error feedback) (Fig. 1A). At 2.6 s, the trial was ended, the screen was blanked, the data saved, and participants would briefly wait for the next trial to begin. Reaction times were not stressed in this experimental procedure. On trials in which the reaction time exceeded 2.6 s, the trial would end, and the participant would wait for the next trial to begin.

Data collection occurred across 2 days in order to observe both the initial learning (first day) and learning retention (second day). For each day, 120 baseline trials (15 sets of eight trials) were completed without a rotation of the cursor. Following these trials, 320 learning trials (40 sets of eight trials) were completed, wherein a 45° clockwise rotation of the cursor was applied. The baseline and learning trials were completed during one continuous fMRI scan. Following this scan, conditions were restored to baseline (i.e., no rotation of cursor) in a separate scan and participants performed 120 washout trials allowing participants to de-adapt before relearning on the second day. Within each set of eight trials, the white target circle can appear in one of eight locations without replacement. In addition to these VMR-related task components, there were also three 6 min resting-state fMRI scans interspersed prior to, between, and following VMR learning and washout. During these resting-state scans, participants were instructed to rest with their eyes open, while fixating a central cross location presented on the screen. The total testing time was 75 min on each testing day (including setup time) with the baseline and rotation scan taking ~30 min, and the washout scan lasting ~9 min (seeSupplementary Materialsfor breakdown of this timing). For our analyses, we only focused on the first day of testing as we were concerned with the initial learning and adaptation.

### 2.3 Behavioural processing

The absolute angle error between the target and final cursor position (RE), and the time taken to make a response (RT) were measured for each trial (Fig. 1B). The angle error was measured as the angle between the initiated direction and the optimal path to the target where positive values mean that the initiated direction was clockwise to the optimal path, and negative values mean that the initiated direction was anticlockwise to the optimal path. To ignore the direction of the error and only consider the amplitude of the error, we took the absolute value of the angle error. Based on the distribution of RT, response times greater than 2 s and less than 0.3 s were removed (the latter was removed due to not being reproducible—could be due to anticipation) (Fig. 1B). RE greater than 100 were removed and could be due to participants sending the cursor in the wrong direction (Fig. 1B). Corresponding fMRI data related to the removed trials were also excluded. For each participant, their RT was plotted against RE to visually demonstrate learning.

The RT and RE of each trial were z-scored and combined by addition to create a Balanced Integration Score (BIS) (Liesefeld & Janczyk, 2019;Liesefeld et al., 2015) (Fig. 1C). Compared to the original BIS score, which measures the number of correct responses, our dataset tracked the angle error (distance of cursor from centre of the target). Therefore, rather than subtracting RE from RT, we have added them together. Note that the BIS score was flipped (multiplied by -1) to make interpretation more intuitive, wherein higher scores are indicative of better performance in the task (and*v.v.*for lower scores). The BIS score was calculated across all trials and conditions to retain between-condition effects. If BIS was calculated per condition, each condition would have the same mean value of 0, therefore eliminating any significant effects between conditions (Liesefeld & Janczyk, 2019). In our study, the participants learned at different rates and reached different levels of “expertise” in the task. To control for this variation, we only included participants who reached a final performance above a certain threshold (BIS ≥ 0), where the final performance was defined as the average BIS score across the final set of eight trials during the washout scan (Fig. 1C). Performance during washout was used to control for participants who learned during the rotation condition but were unable to de-adapt when the task rules were reset. To make sure that the thresholding captured participants who adapted to both the rotation and washout conditions, the BIS scores across trials for each subject were visualised. For the rest of this article, we separated the trials by their condition; for example, Baseline condition has no rotation on the cursor, Rotation includes trials with the 45° rotation, and Washout includes trials with no rotation after the learning (Rotation) trials.

To compare differences between early and late performance within each condition, the mean BIS score was calculated for the first (early) and last (late) sets of eachtaskcondition (Baseline: Sets 1 & 15; Rotation: Sets 16 & 55; Washout: Sets 56 & 70) for each participant. For each condition, a paired-sample t-test was used to compare participants’ early performance against their performance in the last set of trials (Set 1 vs. Set 15; Set 16 vs. Set 55; Set 56 vs. Set 70). While we are using an adaptation task, it is still important to check that task difficulty is similar across conditions, otherwise we would be unable to rule out differences due to cognitive load. To compare task difficulty, a one-way ANOVA was used to compare final performance across the three conditions (Set 15 vs. Set 55 vs. Set 70).

### 2.4 Imaging acquisition

Participants were scanned using a 3T Siemens TIM MAGNETOM Trio MRI scanner located at the Centre for Neuroscience Studies, Queen’s University (Kingston, Ontario, Canada). For each participant on each day, a T1-weighted ADNI MPRAGE anatomical (TR = 1,760 ms, TE = 2.98 ms, field of view = 192 mm × 240 mm × 256 mm, matrix size = 192 × 240 × 256, flip angle = 9°, 1 mm isotropic voxels) was collected. fMRI volumes were acquired using a 32-channel head coil and a T2*-weighted single-shot gradient-echo echo-planar imaging acquisition sequence (TR = 2,000 ms, slice thickness = 4 mm, in-plane resolution = 3 mm × 3 mm, TE = 30 ms, field of view = 240 mm × 240 mm, matrix size = 80 × 80, flip angle = 90°), and acceleration factor (integrated parallel acquisition technologies) = 2 with generalised auto-calibrating partially parallel acquisitions reconstruction. Each volume comprised 34 contiguous (no gap) oblique slices acquired at a 30° caudal tilt with respect to the plane of the anterior and posterior commissure, providing whole-brain coverage of the cerebrum and cerebellum. For each resting-state scan, 180 imaging volumes were collected. For the baseline and learning epochs, we collected a single, continuous scan of 896 imaging volumes. For the washout scan, we collected one scan of 256 imaging volumes. Each scan included an additional eight imaging volumes at both the beginning and the end. All imaging and behavioural data are publicly available in an OpenNeuro repository (Standage et al., 2023, accession number ds004021).

### 2.5 Pre-processing

Neuroimaging data were in BIDS format and were pre-processed with fMRIprep version 21.0.2 (https://fmriprep.org/en/stable/#), a standard pipeline that incorporates toolboxes from the gold-standard preprocessing software in the field.*fMRIPrep*involves the basic pre-processing steps (co-registration, normalisation, unwarping, noise component extraction, segmentation, skull-stripping etc.) and produces a report for quality checking at each step. SeeSupplementary Materialsfor a full description of each step. Regression of 12 head motion artifacts, and the average combined whiter matter, CSF signal was conducted using custom python scripts, with a high-pass filter set at 0.01. Mean BOLD signal time-series data were extracted from the fMRI data for 400 Schaefer cortical regions (Schaefer et al., 2018), 28 cerebellar regions (SUIT atlas;Diedrichsen, 2006), and 26 basal ganglia regions (Tian et al., 2020) using custom python scripts. The following atlases were chosen as robust parcellations that reveal meaningful neurobiological features, with parcels that cover regions from the cerebral cortex, subcortex, and cerebellum which we believe to be involved in expertise and adaptation (de Reus & van den Heuvel, 2013;Diedrichsen, 2006;Khilkevich et al., 2018;Schaefer et al., 2018;Tian et al., 2020). All code is available onhttps://github.com/ShineLabUSYD.

### 2.6 General linear model design

Due to the thresholding of behavioural data in Methods 2.3 (0.3 s < RT < 2 s; RE < 100), we only considered BOLD data with corresponding behavioural data that passed this thresholding. If the behavioural data did not pass these criteria, then the BOLD data of the trial were removed. Due to this thresholding, there was an unequal number of trials per participant.

For each of the remaining trials, we have the timing for when the trial begins (onset time). Following event-related fMRI designs, the onset time for each trial was convolved with the hemodynamic response function (HRF) to approximate the blood flow during a trial (Chee et al., 2003;Friston et al., 1999). In the original dataset, there was no jittering of onset time (consistent trial timing) and the amount of time between trials was less than 10 s, hence there was a consistent overlap of convolved onset times between adjacent trials resulting in temporal blurring of the BOLD activity. In order to guarantee that the BOLD information of each trial was independent of each other trial, we adopted a finite impulse response (FIR) model to select non-overlapping estimates of trial-by-trial BOLD response (Bai et al., 2007;Glover, 1999). Specifically, by taking advantage of the fact that for each trial the behavioural data were thresholded to 2 s (see Methods 2.3 for rationale) and that the imaging sequencing captured BOLD data every 2 s (TR = 2 s), we can select a single imaging volume from the time-series that captured BOLD activity during the task response. Aligning the BOLD time-series to the convolved onset time allowed for the selection of a single imaging volume per trial that contained independent task-related information and took into consideration the delay in activity response as estimated by the HRF (imaging volume at the peak of the convolved onset time) (Fig. 1D).

This collapsed time-series data consisting of peak BOLD measurement was used for the rest of the analyses. Due to the previous removal of trials, the number of remaining trials per participant were unequal. To normalise the number of trials per participant, from each set of eight trials, four trials that met the criteria above were randomly sampled without replacement, resulting in a total of 280 trials per participant across baseline, rotation, and washout. To account for variation in results due to random sampling, we conducted 100 iterations of the random sampling. The mean and standard deviation for cortical, basal ganglia, cerebellar time-series, and BIS were plotted to check for large differences between iterations. The first iteration was chosen as the sample dataset for future analyses.

A general linear model (GLM) was fitted with BIS score as the explanatory variable, and the peak BOLD time-series as the outcome variable. This GLM was fitted for each ROI time-series (400 cortical, 28 cerebellar regions) of each participant as a 1st-level analysis. For group-level analysis (2nd level), significance testing for each region was calculated via a one-sample t-test. Group-level results were calculated for the BIS score and visualised on the brain.

ROI timeseries ~ BIS

A similar GLM was constructed using RT and RE instead of BIS for the explanatory variables. Group-level analysis results were generated via a one-sample t-test and visualised on the brain. Participant-level beta coefficients for RT and RE were averaged and correlated against the group average BIS beta coefficients. To check whether the relationships between BIS and brain regions changed between conditions, a GLM was fitted with BIS divided per condition and the associated peak BOLD time-series. The group average beta coefficients across conditions were correlated against one another. To see whether the BIS brain map was driven by individual participants, the dataset was split roughly in half (n = 12, n = 11). Following a hold-out approach (Dear et al., 2024), by splitting the initial data into smaller sub-datasets (groups) we can evaluate the robustness of the results by seeing how similar the results are across the two groups. Each group had the BIS beta coefficients generated by fitting a GLM with BIS against the peak BOLD time-series (as done above), and the group-level significant testing for each region conducted via a one-sample t-test. The similarity of the thresholded brain map for each group was compared against one another using Pearson’s correlation. To check whether these results are consistent across scales, these analyses were also reproduced in a finer parcellation which consists of the 1,000 Schaefer cortical regions, 28 cerebellar regions, and 26 basal ganglia regions. All comparisons were calculated using Pearson’s correlation, and significance of all correlations was evaluated through spin-permutation testing for cerebral cortical regions.

ROI timeseries ~ RT+RE

### 2.7 Comparison with Neurosynth database

The initial GLM results were surprising as we did not expect primary motor regions to decrease in BOLD activity with performance. As an exploratory analysis and to check whether the BIS brain map was similar to what has been reported in the literature, we compared the BIS brain map against probabilistic fMRI atlases from the neurosynth database version 7 (https://github.com/neurosynth/neurosynth-data). Neurosynth is a meta-analytic tool that synthesises results from more than 15,000 published fMRI studies and generates brain maps where for each voxel, there is an estimated probability that a given cognitive process is associated with activation at that voxel across the studies. The Neuroimaging Meta-Analysis Research Environment (NiMARE;Salo et al., 2022) was accessed, and whole-brain probabilistic maps for 400 neurosynth topics containing terms clustered via a latent Dirichlet allocation (LDA) topic model were downloaded and extracted using custom python scripts. The Pearson’s correlation was used to measure the similarity of the BIS brain map with each of the 400 neurosynth topics. Each topic was then ranked based off how similar or dissimilar they were to the BIS brain map. It is important to note that while the neurosynth database can reproduce robust meta-analytical maps, the selection criteria of studies using topics/terms results in a broad collection of studies which may or may not be comparable with the specific task analysed in this study.

### 2.8 K-means clustering and inter-trial correlations

Individual performance and learning were heterogenous across participants. K-means clustering—a non-supervised clustering algorithm (Hartigan & Wong, 1979)—was used to cluster the participants into distinct sub-groups based off their BIS scores across all conditions. Using BIS scores across all conditions allowed us to group participants by overall trends in performance and not be driven by standout performance in a specific condition. We ran*k*-means clustering from*k*= 2–22 for 100 iterations per level,*k*. To check for cluster similarity across iterations, the adjusted mutual information (AMI;Vinh & Epps, 2009;Vinh et al., 2009,^2010^) was calculated at each*k*. The smallest number of clusters that had the highest AMI across iterations was used for further analyses (*k*= 3).

From differential learning literature, participants who attempt different strategies during learning demonstrate better performance (Schollhorn et al., 2012). In our dataset, for each trial we have a static snapshot of the peak BOLD response in the brain for each trial. Using these data, the average BOLD response was calculated for each cluster. To check whether participants were repeating the same action or trying different strategies, we correlated the average BOLD response for each individual trial with every other trial for the baseline and rotation conditions. To compare strategies between clusters, a two-sample Kolmogorov-Smirnov test was used to determine whether the distribution of correlation values between the clusters came from the same sample.

### 2.9 Brain-state displacement and the energy landscape

In order to evaluate inter-trial changes in BOLD activity, we utilised an approach introduced inMunn et al. (2021)(code for this analysis availablehttps://github.com/ShineLabUSYD/Brainstem_DTI_Attractor_Paper). The energy landscape analysis estimates the likelihood that a given brain state will change to another distinct brain state within a given time window. Changes between brain states are calculated using the mean-squared displacement (MSD; the mean squared difference between 2 periods of time), with greater differences between brain states resulting in a higher MSD value. We then estimate the probability distribution of MSD values and invert this value to estimate the energy required to change from one brain state to another.

In our study, we calculated the energy landscape during the rotation condition for each group cluster separately. For each cluster, we calculated the energy landscape during the first half of the rotation trials and the second half of the rotation trials. The squared displacement of BOLD activity was calculated across all regions between all trials (t_0_to t_0_+ t) within a given window (first half, second half). We then averaged across regions resulting in a single MSD value per trial in the window, < >_r_is the mean across regions. To control for bias towards specific trials, we calculate the MSD of BOLD activity at one trial against all other trials within the window. As our dataset involves learning, to control for temporal structure and learning effects we randomly shuffle the order of trials within a window 100 times (100 permutations) and combine the MSD calculations for all permutations together.

$$MSD_{t,t_{0}}={\langle {\left|x_{t_{0}+t}-x_{t_{0}}\right|}^{2}\rangle }_{r}$$

We then estimated the probability distribution of MSD values, P(MSD_t_), between 0 and 10 within the given window using a Gaussian kernel density estimation.

$$P(MSD_{t})=\;\;\frac{1}{0.1\;\;n}\;\;\sum_{i=1}^{n}\;K\left(\frac{MSD_{t,t_{0}(i)}}{0.1}\right)$$

This probability distribution was then converted to energy,*E*, by taking the negative natural logarithm.

$$E=-ln\left(P(MSD_{t})\right)$$

From this approach, we can describe the likelihood of changes in BOLD activity through the intuition of energy requirements. Highly probable relative changes in BOLD (calculated by MSD) corresponds to a low energy requirement (i.e., small E), and an unlikely change in BOLD has higher energy requirements (i.e., large E) (Munn et al., 2021).

### 2.10 Recurrent neural network modelling

For the first 10 timesteps of the task (corresponding to 100 ms), the direction-selective input neurons are active, providing the network with information of the start direction. Throughout the first 10 timesteps, and also the subsequent 60-timestep delay period, a “task context” input is either active (equal to 1) or inactive (equal to 0), signalling to the network that the context is to rotate or not rotate the input, respectively. After the first 70 timesteps, all inputs turn off, and instead a “go cue” input turns on (equals to 1), signalling to the network that it is time to output the direction. This go cue period persists for 100 timesteps, after which the trial is finished.

Target outputs: in the no rotation context, simply reproduce the input direction. In the rotation context, reproduce the input direction +90°. For training, only the output of the network during the last 100 timesteps (when the go cue comes on) is used in the loss function.

The dynamics of simulated neurons for the recurrent neural network were governed by the standard continuous-time RNN equation and discretised using the Euler method.

$$\tau \frac{dx}{dt}=-x+W^{rec}f(x)+W^{in}I+b$$

In this study, tau was set at 100 ms. The firing rate of each neuron, f(x), was related to its total input x through a rectified*tanh*nonlinearity, f(x) = max(0, tanh(x)). All RNNs in this paper contained 100 recurrent units, with the results being largely insensitive to network size. Each of the 100 neurons in the RNN received input from all other neurons through the recurrent weight matrix W^rec^and also received external input, I(t), through the weight matrix W^in^. Firing rates were linearly combined to produce the output y(t) according to y = W^out^* f(x). The task input and distractor (Fig. 6) were modelled with 100 direction-selective neurons, as described byTeich and Qian (2003); the outputs were modelled with sin and cos functions. During training, all directions (0–360°) were used for the input and distractor.

With the following architecture, three networks were trained with backpropagation initialised from a random initialisation and optimised for 3,000 training steps with Adam in Pytorch, with a learning rate of 0.0001. Networks were trained with square loss.

$$W^{in}=\frac{N(0,1)}{\sqrt{N_{neurons}}},\;W^{rec}=\frac{N(0,1)}{\sqrt{N_{neurons}}},\;W^{out}=0$$

Two of the networks were trained either on a baseline condition (no rotation) or rotated condition (90° rotation). The third network was trained equally on both the baseline and rotation conditions, with the designated condition randomly sorted during training. Task condition was controlled by the “task context”.

The energy landscape was calculated from the outputs of the networks, considering all timesteps leading to the go cue (first 70 timesteps of the task—direction input and first delay). The output signal was two-dimensional (sin and cos). MSD was calculated at adjacent timesteps. The MSD of all trials in a given task condition was concatenated to obtain the final probability mass function. This probability was converted to energy by taking the natural logarithm of the inverse probability.

## 3 Results

### 3.1 Behavioural data

**Fig. 2.**
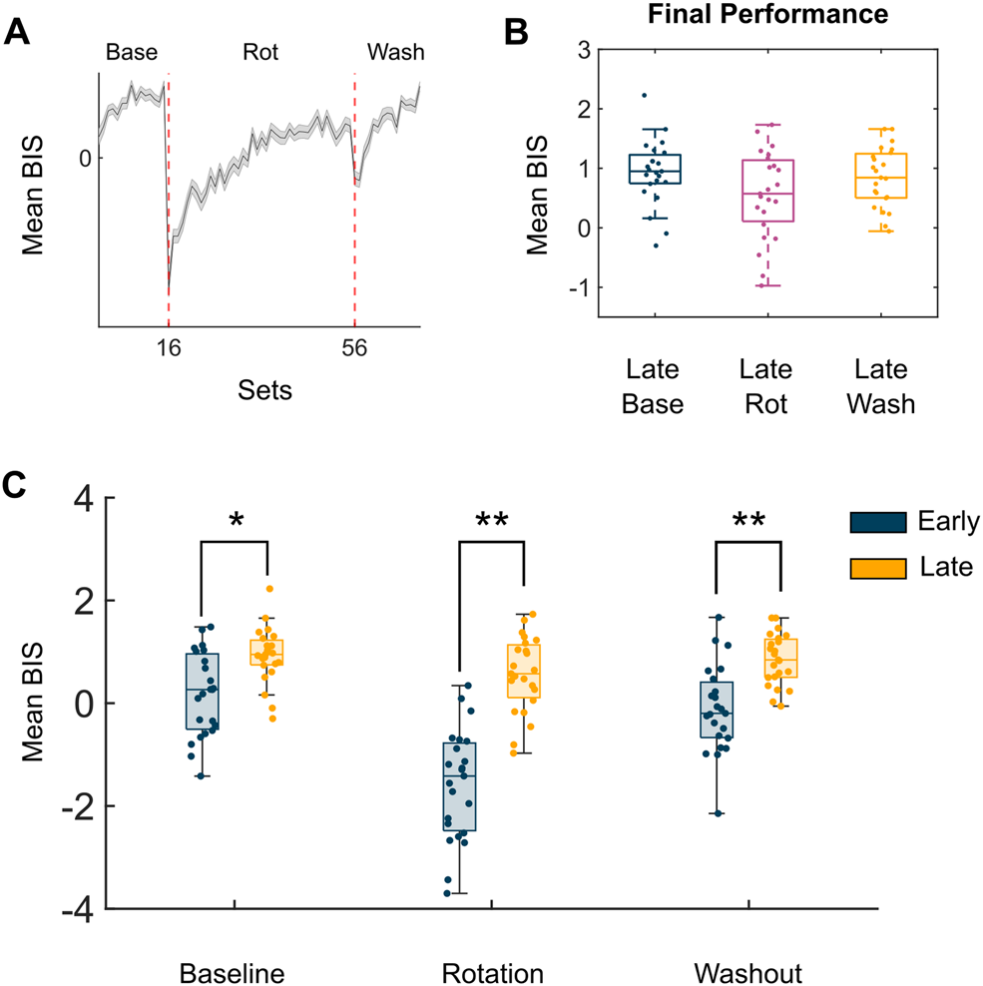
Comparison of behavioural scores within and across conditions. (A) Mean balanced integration score (BIS) across 100 random samples of the data. Standard deviation is plotted as the shaded error bar. Dotted red lines indicate the start of a new condition (16 = rotation, 56 = washout). Base = Baseline, Rot = Rotation, Wash = Washout. (B) Boxplots of BIS scores for participants during the final set of trials (n = 4) for each condition. (C) Boxplots of BIS scores for participants during the first and final set of trials for each condition. Blue boxplots are for the first set, and orange boxplots are the final set. *Notes a significant different p < 0.05, **Notes a significant difference p < 0.01.

To check whether the thresholding of our cohort appropriately selected participants who improved during the task, we compared performance both within and across the three task conditions (baseline, rotation, and washout). There was no significant difference between final performance across conditions from the one-way ANOVA (F = 2.39; p = 0.099;Fig. 2B). Using paired-sample t-tests, we then compared changes in performance from the first and last set of each condition (Baseline = Set 1 vs. Set 15; Rotation = Set 16 vs. Set 55; Washout = Set 56 vs. Set 70). During all conditions, the participants demonstrated significant improvement in performance. In baseline, there was an average increase of 0.755 in BIS score (t = -4.366; p = 0.002;Fig. 2C). During the rotation condition, participants demonstrated the most improvement with an average increase of 2.147 in BIS score (t = -10.956; p < 0.001;Fig. 2D), and during washout there was a significant increase of 0.963 in BIS score (t = -6.342; p < 0.001;Fig. 2E). These results demonstrate that any differences in neural correlates related to performance were due to learning and not differences in task difficulty.

### 3.2 Improved performance is associated with recruitment of cerebellar and basal ganglia regions

**Fig. 3.**
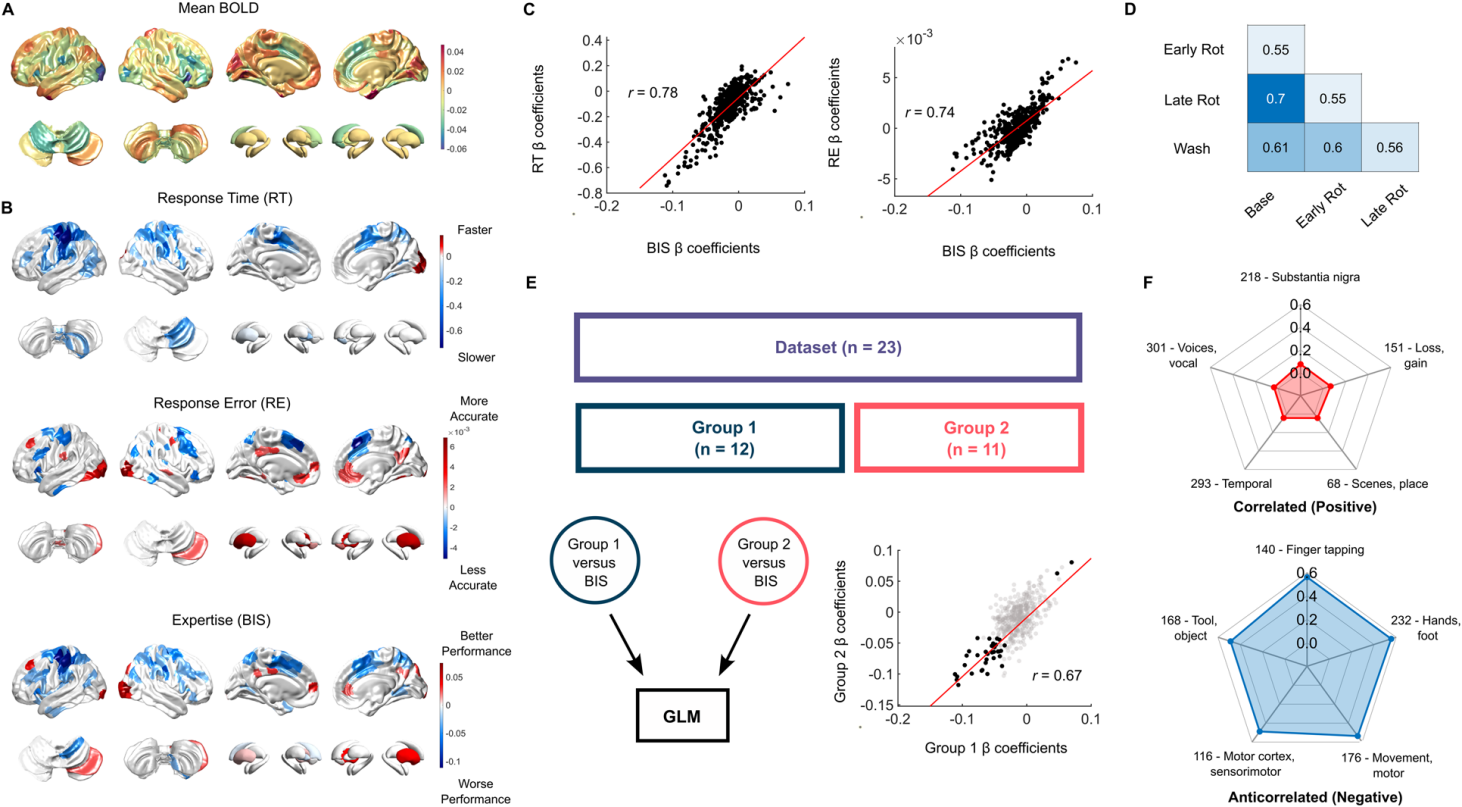
Summary of findings and validation of brain maps from general linear modelling. (A) Average BOLD activity across all participants. (B) Thresholded 2nd-level group beta maps from general linear models for response time (RT), responses error (RE), and the balanced integration score (BIS). (C) Pearson’s correlation comparing the beta coefficients of BIS against RT and RE separately. (D) Pearson’s correlation of BIS beta coefficients across conditions, where rotation has been separated in half into two sub-conditions (baseline, early rotation, late rotation, washout). (E) Validation of individual bias by splitting the dataset into two groups and correlating the beta coefficients for BIS of each group. (F) Top 5 positive and negative correlations of the BIS brain map with the*Neurosynth*LDA-400 topics.

A group average was calculated from the peak BOLD response for all participants (n = 23) across all trials (n = 280) and visualised on a 3D surface (Fig. 3A). During the task, regions that produced a positive BOLD response included sensory areas such as the primary motor, somatosensory, and extrastriate cortices. Control regions such as the medial frontal lobe, temporoparietal junction, inferior temporal lobe, and posterior cingulate cortices, as well as regions from the right Crus I and II, and left semilunar lobule from the cerebellum (Fig. 3). Regions that had a negative BOLD response included sensory regions from the primary visual, lateral motor, and lateral somatosensory cortices. Other regions that produced a negative BOLD response included the inferior frontal lobe and medial prefrontal cortex. There was also a negative BOLD response in lobules I–V of the cerebellum, as well as the head of the caudate nuclei and nucleus accumbens in the basal ganglia.

After fitting the peak BOLD response to BIS in a general linear model (GLM), a one-sample t-test was used to create a thresholded group map (Fig. 3B, bottom panel; p < 0.05). Regions that increased in BOLD with improved performance included the primary visual cortex, right Crus I of the cerebellum, and the right putamen. Regions that were associated with worse performance included regions from premotor, primary motor, and somatosensory cortices, the superior parietal lobe, ventrolateral prefrontal cortex, head of the caudate nuclei, and lobule V on the right hemisphere of the cerebellum. The following results suggest that better performance was mainly driven by increased BOLD in basal ganglia and cerebellar regions, accompanied by a decreased dependence on primary motor and somatosensory regions. Refer to Table 1 in theSupplementary Materialsfor a detailed breakdown of the significant regions and their coefficient estimates from the GLM model.

### 3.3 Balanced integration score retains features of both response time and response error

The BIS group map was compared to group maps fitted with RT and RE in the GLM. From these maps, the regions that were associated with worse performance in the BIS map were due to participants taking longer to respond (Fig. 3B, top panel), and regions that were associated with better performance were mainly driven by participants becoming more accurate with the task (Fig. 3B, middle panel). Furthermore, calculating the correlation between beta coefficients for RT and RE against BIS, there were strong anticorrelations between the beta coefficients (Fig. 3C; r = -0.78, p < 10^-10^; r = -0.74, p < 10^-10^, respectively). These results demonstrate that the BIS score successfully captured the key aspects from RT and RE and is a useful measure of expertise. The BIS scores and BOLD responses were divided into four conditions (baseline, early rotation, late rotation, and washout). Each condition was fit to the peak BOLD response in a GLM. The beta coefficients for each condition were then correlated against the other conditions. All conditions were strongly correlated to each other, with the correlation between baseline and late rotation having the strongest correlation (Fig. 3D; r = 0.7, p < 10^-10^). All brain map correlations underwent spin-permutation testing for the cortical regions, and all comparisons were significantly different (p_spin_< 2 x 10^-5^).

### 3.4 Expertise brain maps are robust to individuals and parcellation effects

After dividing the dataset in half and fitting each group to their expertise scores in two separate GLMs, the average beta coefficients were compared between the two groups. The beta coefficients of the two groups were strongly correlated (Fig. 3E; r = 0.67, p < 10^-10^), providing evidence that these results were not driven by individual participants. To control for parcellation effects (Arslan et al., 2018), we reproduced the above analyses using a finer parcellation that consisted of 1,000 Schaefer cortical nodes, 28 SUIT cerebellar regions, and 26 basal ganglia regions. From these analyses, the results were similar to the current results. For more details on these analyses, refer to theSupplementary Materials.

### 3.5 Expertise brain maps are anticorrelated with motor maps derived from meta-analyses

We generated an expertise (i.e., BIS) brain map from performance in a visuomotor rotation task and compared this brain map against 400 topics from the*Neurosynth*database (Salo et al., 2022;Yarkoni et al., 2011). From these comparisons, the top 5 strongest correlated topics were the*“substantia nigra”*(218),*“loss/gain”*(151),*“scenes/places”*(68),*“temporal”*(293), and*“voices/vocal”*(301). While these topics were the strongest positive correlations, they were relatively weakly correlated (r < 0.2;Fig. 3F, top panel). The top 5 anti-correlated topics were*“finger tapping”*(140),*“hands/foot”*(232),*“movement/motor”*(176),*“motor cortex/sensorimotor”*(116), and*“tool/objects”*(168). In contrast to the positive weights, these topics were strongly anti-correlated (r > 0.5;Fig. 3F, bottom panel). Overall, the*Neurosynth*comparisons suggest that the BIS map generated is not similar to regions mentioned in the literature and is, in fact, opposite to regions described in the majority of motor task studies.

### 3.6 K-means clustering on performance reveals three distinct performance groups

**Fig. 4.**
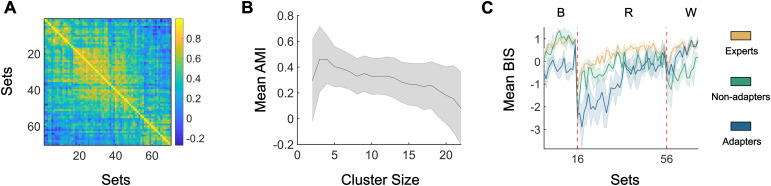
K-means clustering generated three distinct groups. (A) Correlation of the group average balanced integration score (BIS) between sets. (B) Mean and standard deviation (shaded error bar), of the adjusted mutual information (AMI) cluster sizes 2–22 across 100 iterations. (C) Cluster average BIS scores across sets. The yellow line denotes individuals that always performed well (experts), green line for individuals’ performance that worsened across conditions (non-adapters), and blue line for individuals that improved across conditions (adapters).

As an overview for how performance as a whole changed throughout the task, we correlated the group average BIS scores across sets (Fig. 4A). From these correlations, it was apparent that performance in baseline could predict performance during rotation; however, it did not predict performance during washout. To better understand these results, we applied k-means clustering on the BIS scores to check for any distinct sub-groups and calculated the adjusted mutual information (AMI) to select an appropriate number of clusters. From checking the AMI between iterations for each cluster size,*k*= 3 (Fig. 4B) was the smallest number of clusters that were robust across iterations. Our dataset can then be characterised by three clusters (Fig. 4C): a major cluster (experts; n = 16) characterised by high performance across all conditions and two minor clusters, one consisting of participants who had worse performance with each change in task condition (non-adapters; n = 3) and one consisting of participants who started out the worst but improved and demonstrated similar performance to the expert cluster during washout (adapters; n = 4). While there were a small number of participants in the two minor clusters, the number of clusters (*k*= 3) has been chosen after generating 100 iterations for values of from*k*= 2 to*k*= 22 and finding the cluster size with the highest similarity across iterations (AMI). These results suggest that the two minor clusters drive the anticorrelated performance between trials for baseline and washout, as these two groups demonstrated opposite trajectories in performance across task conditions.

### 3.7 Expert adaptation is driven by variation in BOLD activity

**Fig. 5.**
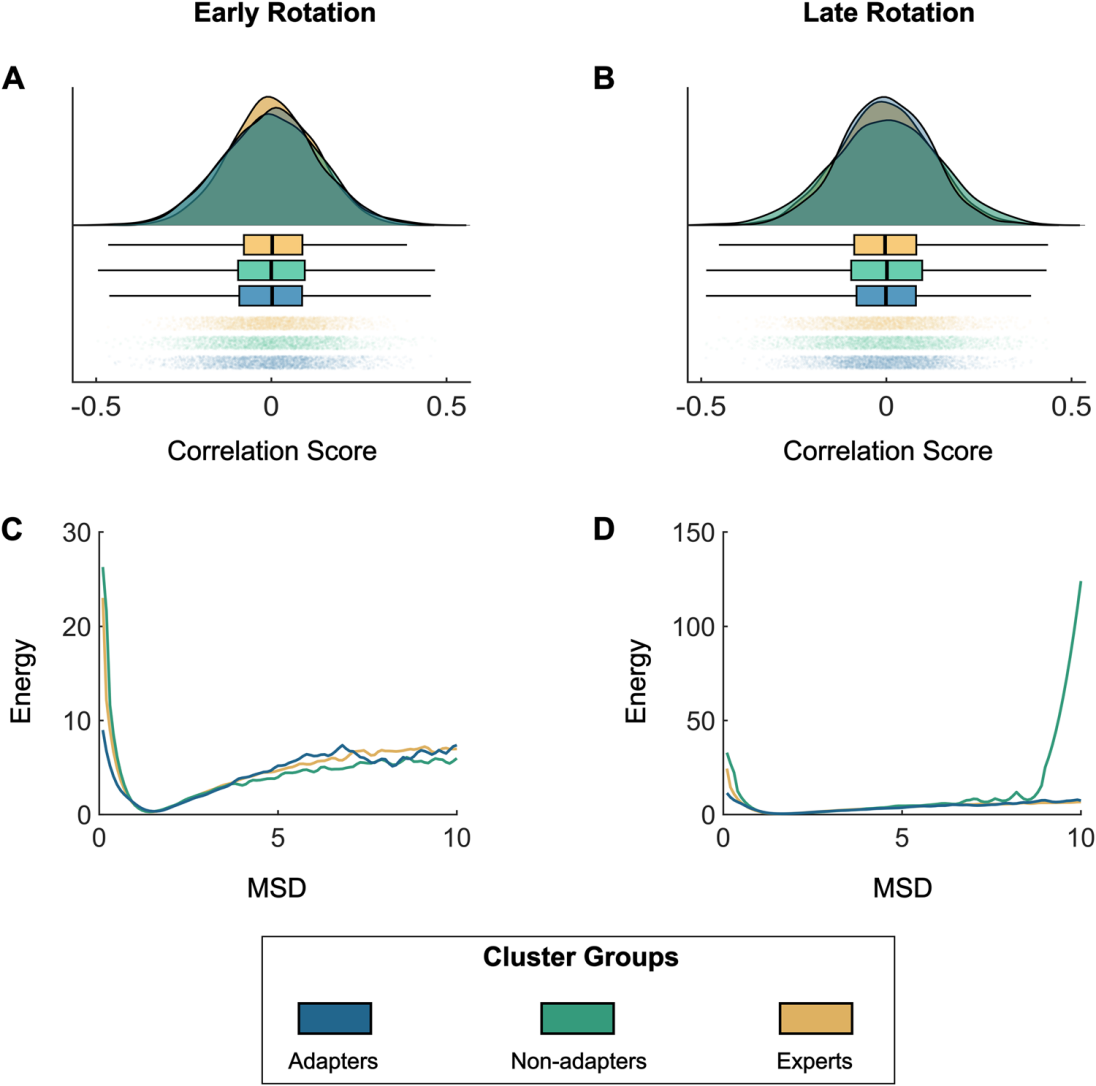
Inter-trial variability analyses for early and late rotation trials. Top panel (A, B) are raincloud plots of inter-trial correlations within the early and late rotation conditions for each group cluster. Bottom panel (C, D) are plots of the energy landscape analyses for each group cluster. Mean-squared displacement (MSD) is calculated between all trials within a condition (early or late rotation). The MSD is converted to Energy by taking the natural logarithm of the inverse probability distribution of MSD.

A possible explanation for the experts and adapters performing well during washout, that is, returning to non-rotated/baseline trials, is that their understanding of the task is comprehensive and robust to small perturbations. To achieve this performance, we suggest that during task learning these groups attempt various distinct actions. This variation would then result in a more flexible, comprehensive understanding of the task. To test this idea, we calculated the correlation between brain patterns across trials during the first and second half of rotation trials (Fig. 5A, B). During the first half, the distribution of correlations for the expert had a higher peak at the zero-correlation, meaning that they produced more unique brain patterns for each trial compared to the other two groups (D_1v2_= 0.0494, p_1v2_= 0.0009; D_1v3_= 0.0484; p_1v3_= 0.0001, respectively). When comparing the distributions of adapters and non-adapters, there was no significant difference in their distributions across the first half of rotation trials (D = 0.0247; p = 0.287). During the second half, the distribution of inter-trial brain map correlations had a higher peak at the zero-lag correlation, similar to the experts (D = 0.0285; p = 0.151), and the distribution of the adapters was also significantly different from the non-adapters (D = 0.0636; p < 0.0001).

Another perspective on flexibility of brain states is attained through energy landscape analysis. In this framework, we ask how much energy is required (inverse of likelihood) for an event to occur. For this study, we calculate how much energy is required to make changes in brain states for each cluster separately. During the first half of rotation trials, all groups demonstrated low energy requirements across brain state changes (Fig. 5C). During the second half, the experts and adapters stayed with this flexible mindset with low energy requirements for both small and large brain state changes (Fig. 5D). However, the non-adapters had large energy requirements for large changes in brains states—they were less likely to make large changes between trials, suggesting a more rigid performance (Fig. 5D). To account for possible temporal and learning biases and the small number of participants in the minor clusters, 100 iterations of the data were generated by randomly shuffling the trials 100 times. Together, these results suggest that expert adaptation is attained by having greater diversity in actions and a willingness to change during learning.

### 3.8 Neural networks trained under multiple conditions develop generalised task performance

Recurrent neural networks (RNN) offer a unique means to explore computational strategies employed by an organism to solve a given behavioural task whereby the complete space of algorithmic solutions can be constrained by the investigator to assess biological validity of implementation within a particular organism, but also the versatility of solutions as a function of environmental variables. Here, we exploit this degrees-of-freedom control to demonstrate potential computation strategies being employed in an RNN trained on a simplified rotation paradigm analogous to the visuomotor rotation task. We train three separate RNNs under unique conditions, compare their performance as a function of changes to input/environmental variables to determine each solution’s flexibility, and demonstrate network features comparable to those seen in the imaging data during the visuomotor rotation task.

**Fig. 6.**
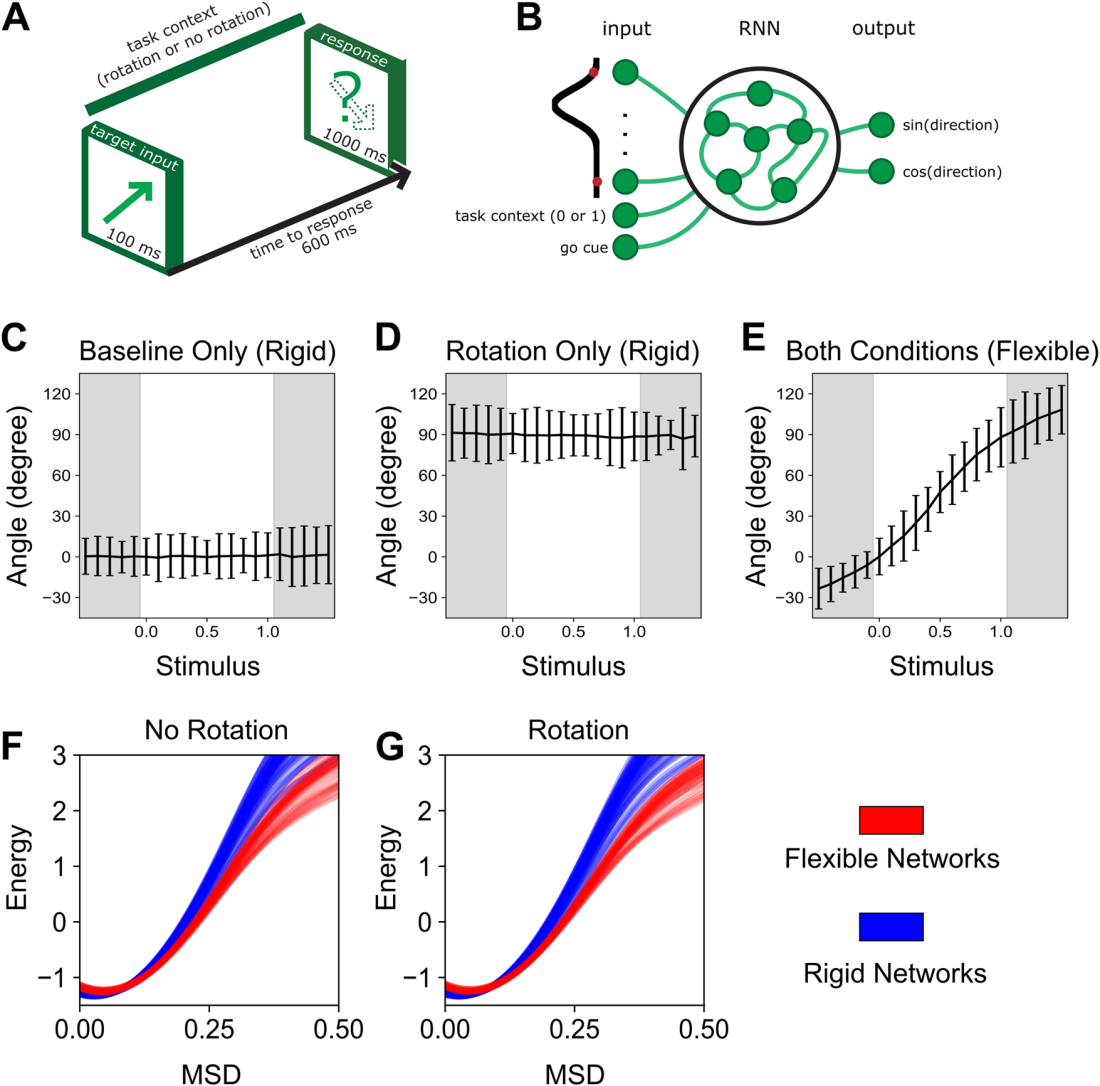
A recurrent neural network model of sensorimotor adaptation. (A) Continuous time recurrent neural networks (RNN) were trained to output either the same direction as the input (context = 0) or a 90° rotation of the input (context = 1). (B) Architecture of the RNN. (C, D, E) Following training, the networks were tested across a range of task contexts (0 to 1) which varied the amount of rotation applied on the input. (C, D) The two networks that were trained only on baseline (0) or a 90° rotation (1) only generated the trained output (rigid networks). (E) The network trained on both baseline and rotation performed well under both conditions as well as for all angles of rotation between 0 and 90° (flexible network). (F, G) For each of the three original networks, 20 additional networks were trained under the same conditions with random seeds. After training, for each network the MSD was calculated across adjacent trials and the probability distribution was estimated and converted into energy. For both the No Rotation and Rotation conditions, the networks trained on both conditions (flexible networks) had lower energy requirements than the networks only trained on one condition (rigid networks) across higher MSD values.

Two of the networks were trained on only one task condition (baseline or rotation) and developed a rigid solution to the task, specific to only those task conditions seen during training (Fig. 6A, B). The first network was trained similar to the baseline condition, outputting the input angle for all angles of rotation (0°–90°). The second network trained only on the rotation condition, output the 90° rotated input for all angles of rotation. Next, a third network was trained on both the baseline and rotation conditions. This network developed a more flexible and generalised solution to the task, performing accurately across all angles of rotation between 0° and 90° (Fig. 6C). That is, despite only learning on two environmental conditions, namely non-rotated and 90° rotated conditions, the network learned a more general solution capable of handling any specified rotation angle between 0° and 90° which included environmental conditions the network has not seen.

Finally, we calculated the attractor landscape for each of the networks and, thus, the likelihood of their corresponding activity changes. The third, flexible, network was more likely to change in activity across all MSD values. The energy required to change was lower across all MSD values for the flexible network compared to the rigid networks, which matched with the networks’ variable outputs (Fig. 6D).

## 4 Discussion

In this study, we used a unique measurement of skill expertise to determine the neural signatures of experts in a sensorimotor adaptation task. We demonstrate a novel application of the balanced integration score (BIS) for fMRI studies, providing an alternative perspective on neural signatures of expertise. Expertise in adaptation was associated with decreased recruitment of the primary motor cortex, and increased dependence on cerebellar and subcortical regions. Furthermore, by exploring sub-groups within the dataset, we found evidence that flexibility in the BOLD response was key for rapid adaptation and robustness to perturbations. During the rotation task, participants who learned to adapt were more likely to have large inter-trial differences in brain maps, consistent with behaviour observed in expert individuals. These results contrasted with participants who failed to adapt and were not as robust to perturbations. To complement the fMRI analyses, we trained RNNs to perform a similar task and demonstrated that a network only trained on the baseline condition was unable to adapt to rotation perturbation; however, a network trained on both the baseline and a 90° rotation was competent across all angles of rotation between 0° and 90°. Overall, these results suggest that expertise in adaptation is facilitated by flexible brain patterns that are also dependent on increased BOLD responses from subcortical and cerebellar regions.

The balanced integration score (i.e., BIS) captures the key aspects of both speed and accuracy, serving as an operationalisation for expertise in the sensorimotor adaptation task. There is a well-known trade-off between speed and accuracy, wherein accuracy is improved by being careful and taking more time, whereas working quickly results in more reckless and inaccurate responses (Heitz, 2014). Importantly, we define expertise as having the capability to customise the speed-accuracy trade-off, resulting in performances which are both fast and accurate (Debarnot et al., 2014). While the BIS has previously been applied in mental rotation tasks (Liesefeld et al., 2015), typically neuroimaging studies would either analyse speed and accuracy separately, or estimate a speed-accuracy trade-off function (Heitz, 2014). A key difference between the BIS and the typical speed-accuracy curve estimates is that the BIS retains the raw values whereas speed-accuracy estimates fit a curve to summarise the data, potentially applying a smoothing effect (Heitz, 2014;Liesefeld & Janczyk, 2019). In this study, combining the BIS with fMRI BOLD measurements successfully summarised speed and accuracy with a comprehensive brain map in a sensorimotor adaptation task. Furthermore, these results matched findings from analysing speed and accuracy separately. The comprehensive brain map generated from the BIS scores was also anticorrelated to meta-analytical motor brain maps, supporting studies that suggest expertise in a task is different to the general brain activity required to complete a task (Bassett et al., 2015;Yang, 2015). Therefore, our study provides evidence for the utility of the BIS in neuroimaging studies, generating novel insights of expertise.

Expert performance during adaptation is dependent on the recruitment of subcortical regions, such as the cerebellum and basal ganglia, which is a conclusion that has been underappreciated in existing task-based imaging studies. There are numerous reasons for this, ranging from statistical constraints (e.g., induced head motion while completing a task), to hardware choices (e.g., designing coils that augment signal-to-noise ratios near the cortical surface) and even scanning procedures themselves (e.g., the harrowing choice in many prior studies to completely remove large portions of the cerebellum from the field-of-view). These studies inherently limit their scope to the cerebral cortex, ignoring the extensive brain-wide connections between the cortex, subcortex, and cerebellum (Kelly & Strick, 2003;Middleton & Strick, 1994;Shine, 2021,^2022^). Our study builds upon this literature by trying to understand how interactions between the cortex, subcortex, and cerebellum facilitate skilled performance in a task. Adapting to the final position of the target and not focusing on the trajectory taken has previously been associated with driving learning in visuomotor adaptation paradigms (Anguera et al., 2007). Our results agree with this notion, as better performance was driven by minimising error in the task and this minimisation involved cortical and cerebellar regions. Furthermore, our results found that motor regions in the cerebral cortex and cerebellum were associated with slower responses, providing evidence that decreased dependence on cortical regions is related to higher efficiency in the task (Bassett et al., 2015). By constraining behaviour by both the task and performance, our study demonstrated that expertise in adaptation was driven by both an increased recruitment of subcortical and cerebellar regions and a decreased dependence on motor cortical regions.

The involvement of the cerebellum in adaptation learning aligns with previous ideas, suggesting that the cerebellum provides functions for feed-forward processing (Ramnani & Miall, 2004;Wolpert & Flanagan, 2001) and pattern separation (Cayco Gajic & Silver, 2019). These functions could help disentangle the different interactions between inputs and outputs to the motor cortex, developing an internal model that provides a basis set of principles of possible ways to complete an action (Doyon & Benali, 2005;Egger et al., 2019;Imamizu et al., 2000;Wolpert et al., 1998). These actions would account for changes in the environment, allowing individuals to rapidly adapt to any changes in the task, a key aspect of expert behaviour (Debarnot et al., 2014;Imamizu et al., 2000). An important aspect is the specificity of the task design and how this specificity influences the current results. For example, the current visuomotor paradigm is biased towards visual sensory processes and right-hand activation. Our results reflect this task specificity with lateralisation of cerebellar activity in the right cerebellar hemisphere and recruitment of regions from the visual cortex. The involvement of right cerebellar activity aligns with the general notion that cerebellar regions map contralateral with the cerebrum (Xue et al., 2021). In this case, right-hand actions map to the left cerebral hemisphere which maps to the right cerebellar hemisphere. Primary visual regions are typically recruited during visuomotor tasks (Anguera et al., 2007;Sendhilnathan et al., 2024;Tzvi et al., 2022); however, they are usually not associated with learning (Striemer et al., 2019). In fact, the reliance on visual feedback and explicit strategies has been deemed less efficient compared to implicit learning processes (Mazzoni & Krakauer, 2006). While there are very few visual inputs directly from the primary visual cortex to the cerebellum (Glickstein, 2000;Glickstein & Doron, 2008;Glickstein et al., 1980), it is possible that primary visual activity can interact with cerebellar activity via the pontine nuclei or higher order visual regions (Glickstein, 2000;Tzvi et al., 2020). Finally, the association between the right putamen and higher performance in visuomotor adaptation is possibly related to functional lateralisation in the basal ganglia (Scholz et al., 2000;Viñas-Guasch & Wu, 2017). While it is more common to think about dividing the putamen functionally into anterior and posterior zones (Milardi et al., 2019;Oberhuber et al., 2013), it has been suggested that lateralisation is also a key feature of the putamen with the right putamen serving a role in visual and orthographic processing and the left putamen serving a role in language processing (Viñas-Guasch & Wu, 2017). While the micro-details behind cortical, cerebellar, and subcortical interactions are still not clear, these results highlight the importance of the cerebellum and subcortex in expertise and adaptation.

Adaptation is facilitated by a flexible mindset and a willingness to explore the boundaries of the task (Frank et al., 2008;Henz & Schöllhorn, 2016;Santos et al., 2018). Traditional training plans that focus on repeating the same action “10,000 times” overfit learning to the context of the action, resulting in diminished ability to adapt to changes in the environment. For example, a tennis player who practises serving close to the mid-line (as you do in singles) may struggle to translate this skill to serving in doubles, where they are required to stand closer towards the sidelines. A framework of learning that potentially overcomes this overfitting and facilitates adaptation is differential learning (Schollhorn et al., 2012). Studies that have trained athletes using differential learning demonstrated increased performance in various sports compared to athletes who follow more traditional training regimes (Beckmann & Schöllhorn, 2003;Coutinho et al., 2018;Henz & Schöllhorn, 2016). We suggest that differential learning improves final performance by allowing individuals to explore the boundaries of the action and perform at a high level independent of the context. In our study, while the participants were not instructed specifically on learning strategies, we found that participants who had more variable brain patterns performed better in the final condition. We suggest that variation in brain patterns across trials is due to participants attempting different actions/strategies allowing them to develop a comprehensive understanding of the task. While steps have been taken to account for the low number of participants in the sub-groups, replication of these results in larger sample sizes could further cement the relationship between variation during learning and generalisation across tasks. In addition, when training recurrent neural networks on the same task, the network that learned both the baseline and rotation conditions was able to adapt to intermediate amplitudes of rotation that lie between the baseline and rotation conditions, suggesting a generalisation of skill between the boundaries of the task. Variation in strategies and actions develops flexible learning of a task and generalises performance across different scenarios.

The current study focused on expertise in sensorimotor adaptation, specifically visuomotor rotations and how cortico-cerebellar circuits change throughout learning. However, cortico-cerebellar interactions also exist outside motor regions, specifically connections with granular regions in the frontal lobes of the cerebral cortex (Kelly & Strick, 2003;Ramnani, 2012;Ramnani et al., 2006). With the relatively homogenous cytoarchitecture of the cerebellum (Diedrichsen et al., 2019;Schmahmann & Pandya, 1997) and connections with non-motor regions (Kelly & Strick, 2003;Ramnani, 2012;Ramnani et al., 2006), we anticipate that the cerebellum will undoubtedly play an important role in pattern separation during cognitive tasks. As it stands, apart from motor learning and coordination, cerebellar activity has been found to be integral to a number of higher order functions such as working memory (Baier et al., 2014;Brissenden & Somers, 2019;D’Esposito et al., 1999) and parallel processing (Müller et al., 2023). Future research is required to understand cerebellar contributions to these various behaviours and could provide a parsimonious view on expertise and skilled performance across both motor and cognitive aspects.

This study revealed cortico-cerebellar patterns related to expertise in a sensorimotor adaptation task. Dependence on subcortical and cerebellar regions increased with performance and was accompanied by decreased recruitment of primary motor regions, suggesting a subcortical-cerebellar drive on expertise in adaptation. We also found that variable brain patterns and the willingness to change strategies between trials during learning resulted in improved performance in the final task. By focusing analyses on whole-brain interactions that include subcortical and cerebellar regions, a comprehensive understanding of adaptation across tasks and modalities can be attained.

## Supplementary Material

Supplementary Material

## Data Availability

All imaging and behavioural data are publicly available in an OpenNeuro repository (Standage et al., 2023, accession number ds004021). Analysis of both the behavioural and functional MRI data was conducted in MATLAB. Codes required to reproduce the statistical analyses and figures are publicly availablehttps://github.com/ShineLabUSYD/VM_Adaptation. The recurrent neural network analysis was conducted in python and is available athttps://github.com/azaho/adaptation_rnn.

## References

[b1] Albus , J. S. ( 1971 ). A theory of cerebellar function . Mathematical Biosciences , 10 , 25 – 61 . 10.1016/0025-5564(71)90051-4

[b2] Anguera , J. A. , Russell , C. A. , Noll , D. C. , & Seidler , R. D. ( 2007 ). Neural correlates associated with intermanual transfer of sensorimotor adaptation . Brain Research , 1185 , 136 – 151 . 10.1016/j.brainres.2007.09.088 17996854

[b3] Areshenkoff , C. , Gale , D. J. , Standage , D. , Nashed , J. Y. , Flanagan , J. R. , & Gallivan , J. P. ( 2022 ). Neural excursions from manifold structure explain patterns of learning during human sensorimotor adaptation . eLife , 11 , e74591 . 10.7554/eLife.74591 35438633 PMC9018069

[b4] Arslan , S. , Ktena , S. I. , Makropoulos , A. , Robinson , E. C. , Rueckert , D. , & Parisot , S. ( 2018 ). Human brain mapping: A systematic comparison of parcellation methods for the human cerebral cortex . NeuroImage , 170 , 5 – 30 . 10.1016/j.neuroimage.2017.04.014 28412442

[b5] Bai , B. , Kantor , P. , & Shokoufandeh , A. ( 2007 ). Effectiveness of the finite impulse response model in content-based fMRI image retrieval . In Ayache N. , Ourselin S. , & Maeder A. (Eds.), Medical Image Computing and Computer-Assisted Intervention—MICCAI 2007, Lecture Notes in Computer Science (pp. 742 – 750 ). Springer . 10.1007/978-3-540-75759-7_90 18044635

[b6] Baier , B. , Müller , N. G. , & Dieterich , M. ( 2014 ). What part of the cerebellum contributes to a visuospatial working memory task ? Annals of Neurology , 76 , 754 – 757 . 10.1002/ana.24272 25220347

[b7] Bassett , D. S. , Yang , M. , Wymbs , N. F. , & Grafton , S. T. ( 2015 ). Learning-induced autonomy of sensorimotor systems . Nature Neuroscience , 18 , 744 – 751 . 10.1038/nn.3993 25849989 PMC6368853

[b8] Bastian , A. J. ( 2006 ). Learning to predict the future: The cerebellum adapts feedforward movement control . Current Opinion in Neurobiology , 16 , 645 – 649 . 10.1016/j.conb.2006.08.016 17071073

[b9] Beckmann , H. , & Schöllhorn , W. I. ( 2003 ). Differencial learning in shot put . Presented at the European Workshop on Movement Science, Köln, Germany .

[b10] Berlot , E. , Popp , N. J. , & Diedrichsen , J. ( 2020 ). A critical re-evaluation of fMRI signatures of motor sequence learning . eLife , 9 , e55241 . 10.7554/eLife.55241 32401193 PMC7266617

[b11] Bostan , A. C. , & Strick , P. L. ( 2018 ). The basal ganglia and the cerebellum: Nodes in an integrated network . Nature Reviews Neuroscience , 19 , 338 – 350 . 10.1038/s41583-018-0002-7 29643480 PMC6503669

[b12] Bransford , J. D. , & Schwartz , D. L. ( 1999 ). Rethinking transfer: A simple proposal with multiple implications . Review of Research in Education , 24 , 61 – 100 . 10.2307/1167267

[b13] Brissenden , J. A. , & Somers , D. C. ( 2019 ). Cortico-cerebellar networks for visual attention and working memory . Current Opinion in Psychology , 29 , 239 – 247 . 10.1016/j.copsyc.2019.05.003 31202085 PMC7256875

[b14] Cayco Gajic , N. A. , & Silver , R. A. ( 2019 ). Re-evaluating circuit mechanisms underlying pattern separation . Neuron , 101 , 584 – 602 . 10.1016/j.neuron.2019.01.044 30790539 PMC7028396

[b15] Chee , M. , Venkatraman , V. , Westphal , C. , & Siong Chun , S . ( 2003 ). Comparison of block and event-related fMRI designs in evaluating the word-frequency effect . Human Brain Mapping , 18 ( 3 ), 186 – 193 . 10.1002/hbm.10092 12599276 PMC6871864

[b16] Cheung , J. J. H. , & Kulasegaram , K. M. ( 2022 ). Beyond the tensions within transfer theories: Implications for adaptive expertise in the health professions . Advances in Health Sciences Education , 27 , 1293 – 1315 . 10.1007/s10459-022-10174-y 36369374

[b17] Churchland , M. M. , Cunningham , J. P. , Kaufman , M. T. , Foster , J. D. , Nuyujukian , P. , Ryu , S. I. , & Shenoy , K. V. ( 2012 ). Neural population dynamics during reaching . Nature , 487 , 51 – 56 . 10.1038/nature11129 22722855 PMC3393826

[b18] Churchland , M. M. , Santhanam , G. , & Shenoy , K. V. ( 2006 ). Preparatory activity in premotor and motor cortex reflects the speed of the upcoming reach . Journal of Neurophysiology , 96 , 3130 – 3146 . 10.1152/jn.00307.2006 16855111

[b19] Cohen , O. , Harel , R. , Aumann , T. D. , Israel , Z. , & Prut , Y. ( 2017 ). Parallel processing of internal and external feedback in the spinocerebellar system of primates . Journal of Neurophysiology , 118 , 254 – 266 . 10.1152/jn.00825.2016 28381489 PMC5498734

[b20] Coutinho , D. , Santos , S. , Gonçalves , B. , Travassos , B. , Wong , D. P. , Schöllhorn , W. , & Sampaio , J. ( 2018 ). The effects of an enrichment training program for youth football attackers . PLoS One , 13 , e0199008 . 10.1371/journal.pone.0199008 29897985 PMC5999098

[b21] Debarnot , U. , Sperduti , M. , Di Rienzo , F. , & Guillot , A. ( 2014 ). Experts bodies, experts minds: How physical and mental training shape the brain . Frontiers in Human Neuroscience , 8 , 280 . 10.3389/fnhum.2014.00280 24847236 PMC4019873

[b22] de Reus , M. A. , & van den Heuvel , M. P . ( 2013 ). The parcellation-based connectome: Limitations and extensions . NeuroImage , 80 , 397 – 404 . 10.1016/j.neuroimage.2013.03.053 23558097

[b201] Dear , R. , Wagstyl , K. , Seidlitz , J. , Markello , R. D. , Arnatkevičiūtė , A. , Anderson , K. M. , Bethlehem , R. A. I. , Raznahan , A. , Bullmore , E. T. , Vértes , P. E . ( 2024 ). Cortical gene expression architecture links healthy neurodevelopment to the imaging, transcriptomics and genetics of autism and schizophrenia . Nature Neuroscience , 27 , 1075 – 1086 . 10.1038/s41593-024-01624-4 38649755 PMC11156586

[b23] D’Esposito , M. , Postle , B. R. , Ballard , D. , & Lease , J. ( 1999 ). Maintenance versus manipulation of information held in working memory: An event-related fMRI study . Brain and Cognition , 41 , 66 – 86 . 10.1006/brcg.1999.1096 10536086

[b24] Diedrichsen , J. ( 2006 ). A spatially unbiased atlas template of the human cerebellum . NeuroImage , 33 , 127 – 138 . 10.1016/j.neuroimage.2006.05.056 16904911

[b25] Diedrichsen , J. , King , M. , Hernandez-Castillo , C. , Sereno , M. , & Ivry , R. B. ( 2019 ). Universal transform or multiple functionality? Understanding the contribution of the human cerebellum across task domains . Neuron , 102 , 918 – 928 . 10.1016/j.neuron.2019.04.021 31170400 PMC6686189

[b26] Doyon , J. , & Benali , H. ( 2005 ). Reorganization and plasticity in the adult brain during learning of motor skills . Current Opinion in Neurobiology , 15 , 161 – 167 . 10.1016/j.conb.2005.03.004 15831397

[b27] Doyon , J. , Penhune , V. , & Ungerleider , L. G. ( 2003 ). Distinct contribution of the cortico-striatal and cortico-cerebellar systems to motor skill learning . Neuropsychologia , 41 , 252 – 262 . 10.1016/S0028-3932(02)00158-6 12457751

[b28] Egger , S. W. , Remington , E. D. , Chang , C.-J. , & Jazayeri , M. ( 2019 ). Internal models of sensorimotor integration regulate cortical dynamics . Nature Neuroscience , 22 , 1871 – 1882 . 10.1038/s41593-019-0500-6 31591558 PMC6903408

[b29] Foerde , K. , & Shohamy , D. ( 2011 ). The role of the basal ganglia in learning and memory: Insight from Parkinson’s disease . Neurobiology of Learning and Memory , 96 , 624 – 636 . 10.1016/j.nlm.2011.08.006 21945835 PMC3772079

[b30] Frank , T. D. , Michelbrink , M. , Beckmann , H. , & Schöllhorn , W. I. ( 2008 ). A quantitative dynamical systems approach to differential learning: Self-organization principle and order parameter equations . Biological Cybernetics , 98 , 19 – 31 . 10.1007/s00422-007-0193-x 18026746

[b31] Friston , K. J. , Zarahn , E. , Josephs , O. , Henson , R. N. A. , & Dale , A. M. ( 1999 ). Stochastic designs in event-related fMRI . NeuroImage , 10 , 607 – 619 . 10.1006/nimg.1999.0498 10547338

[b32] Gallego , J. A. , Perich , M. G. , Chowdhury , R. H. , Solla , S. A. , & Miller , L. E. ( 2020 ). Long-term stability of cortical population dynamics underlying consistent behavior . Nature Neuroscience , 23 , 260 – 270 . 10.1038/s41593-019-0555-4 31907438 PMC7007364

[b33] Gilbert , P. F. C. , & Thach , W. T. ( 1977 ). Purkinje cell activity during motor learning . Brain Research , 128 , 309 – 328 . 10.1016/0006-8993(77)90997-0 194656

[b34] Glickstein , M. ( 2000 ). How are visual areas of the brain connected to motor areas for the sensory guidance of movement ? Trends in Neurosciences , 23 ( 12 ), 613 – 617 . 10.1016/s0166-2236(00)01681-7 11137151

[b35] Glickstein , M. , Cohen , J. L. , Dixon , B. , Gibson , A. , Hollins , M. , Labossiere , E. , & Robinson , F. ( 1980 ). Corticopontine visual projections in macaque monkeys . The Journal of Comparative Neurology , 190 ( 2 ), 209 – 229 . 10.1002/cne.901900202 7381057

[b36] Glickstein , M. , & Doron , K. ( 2008 ). Cerebellum: Connections and functions . Cerebellum , 7 ( 4 ), 589 – 594 . 10.1007/s12311-008-0074-4 19002543

[b37] Glover , G. H. ( 1999 ). Deconvolution of impulse response in event-related BOLD fMRI . NeuroImage , 9 , 416 – 429 . 10.1006/nimg.1998.0419 10191170

[b38] Graybiel , A. M. , & Grafton , S. T. ( 2015 ). The striatum: Where skills and habits meet . Cold Spring Harbor Perspectives in Biology , 7 , a021691 . 10.1101/cshperspect.a021691 26238359 PMC4526748

[b39] Hartigan , J. A. , & Wong , M. A. ( 1979 ). Algorithm AS 136: A K-means clustering algorithm . Journal of the Royal Statistical Society. Series C (Applied Statistics) , 28 , 100 – 108 . 10.2307/2346830

[b40] Hayter , A. L. , Langdon , D. W. , & Ramnani , N. ( 2007 ). Cerebellar contributions to working memory . NeuroImage , 36 , 943 – 954 . 10.1016/j.neuroimage.2007.03.011 17468013

[b41] Heitz , R. P. ( 2014 ). The speed-accuracy tradeoff: History, physiology, methodology, and behavior . Frontiers in Neuroscience , 8 , 150 . 10.3389/fnins.2014.00150 24966810 PMC4052662

[b42] Henz , D. , & Schöllhorn , W. I. ( 2016 ). Differential training facilitates early consolidation in motor learning . Frontiers in Behavioral Neuroscience , 10 , 199 . 10.3389/fnbeh.2016.00199 27818627 PMC5073148

[b43] Hikosaka , O. , Nakamura , K. , Sakai , K. , & Nakahara , H. ( 2002 ). Central mechanisms of motor skill learning . Current Opinion in Neurobiology , 12 , 217 – 222 . 10.1016/S0959-4388(02)00307-0 12015240

[b44] Imamizu , H. , & Kawato , M. ( 2009 ). Brain mechanisms for predictive control by switching internal models: Implications for higher-order cognitive functions . Psychological Research , 73 , 527 – 544 . 10.1007/s00426-009-0235-1 19347360

[b45] Imamizu , H. , Miyauchi , S. , Tamada , T. , Sasaki , Y. , Takino , R. , & Kawato , M. ( 2000 ). Human cerebellar activity reflecting an acquired internal model of a new tool . Nature , 403 , 192 – 195 . 10.1038/35003194 10646603

[b46] Kawai , R. , Markman , T. , Poddar , R. , Ko , R. , Fantana , A. L. , Dhawale , A. K. , Kampff , A. R. , & Ölveczky , B. P. ( 2015 ). Motor cortex is required for learning but not for executing a motor skill . Neuron , 86 , 800 – 812 . 10.1016/j.neuron.2015.03.024 25892304 PMC5939934

[b47] Kelly , R. M. , & Strick , P. L. ( 2003 ). Cerebellar loops with motor cortex and prefrontal cortex of a nonhuman primate . Journal of Neuroscience , 23 , 8432 – 8444 . 10.1523/JNEUROSCI.23-23-08432.2003 12968006 PMC6740694

[b48] Khilkevich , A. , Zambrano , J. , Richards , M.-M. , & Mauk , M. D. ( 2018 ). Cerebellar implementation of movement sequences through feedback . eLife , 7 , e37443 . 10.7554/eLife.37443 30063004 PMC6107335

[b49] Kostadinov , D. , & Häusser , M. ( 2022 ). Reward signals in the cerebellum: Origins, targets, and functional implications . Neuron , 110 ( 8 ), 1290 – 1303 . 10.1016/j.neuron.2022.02.015 35325616

[b50] Koziol , L. F. , Budding , D. , Andreasen , N. , D’Arrigo , S. , Bulgheroni , S. , Imamizu , H. , Ito , M. , Manto , M. , Marvel , C. , Parker , K. , Pezzulo , G. , Ramnani , N. , Riva , D. , Schmahmann , J. , Vandervert , L. , & Yamazaki , T. ( 2014 ). Consensus paper: The Cerebellum’s role in movement and cognition . Cerebellum , 13 , 151 – 177 . 10.1007/s12311-013-0511-x 23996631 PMC4089997

[b51] Krakauer , J. W. ( 2009 ). Motor learning and consolidation: The case of visuomotor rotation . In Sternad D. (Ed.), Progress in motor control, advances in experimental medicine and biology (pp. 405 – 421 ). Springer . 10.1007/978-0-387-77064-2_21 PMC2672910 19227512

[b52] Lanciego , J. L. , Luquin , N. , & Obeso , J. A. ( 2012 ). Functional neuroanatomy of the basal ganglia . Cold Spring Harbor Perspectives in Medicine , 2 , a009621 . 10.1101/cshperspect.a009621 23071379 PMC3543080

[b53] Liesefeld , H. R. , Fu , X. , & Zimmer , H. D. ( 2015 ). Fast and careless or careful and slow? Apparent holistic processing in mental rotation is explained by speed-accuracy trade-offs . Journal of Experimental Psychology: Learning, Memory, and Cognition , 41 ( 4 ), 1140 – 1151 . 10.1037/xlm0000081 25528084

[b54] Liesefeld , H. R. , & Janczyk , M. ( 2019 ). Combining speed and accuracy to control for speed-accuracy trade-offs(?) . Behavior Research , 51 , 40 – 60 . 10.3758/s13428-018-1076-x 30022459

[b55] Mazzoni , P. , & Krakauer , J. W. ( 2006 ). An implicit plan overrides an explicit strategy during visuomotor adaptation . Journal of Neuroscience , 26 , 3642 – 3645 . 10.1523/JNEUROSCI.5317-05.2006 16597717 PMC6674132

[b56] McDougle , S. D. , Ivry , R. B. , & Taylor , J. A. ( 2016 ). Taking aim at the cognitive side of learning in sensorimotor adaptation tasks . Trends in Cognitive Sciences , 20 , 535 – 544 . 10.1016/j.tics.2016.05.002 27261056 PMC4912867

[b57] Middleton , F. A. , & Strick , P. L. ( 1994 ). Anatomical evidence for cerebellar and basal ganglia involvement in higher cognitive function . Science , 266 , 458 – 461 . 10.1126/science.7939688 7939688

[b58] Milardi , D. , Quartarone , A. , Bramanti , A. , Anastasi , G. , Bertino , S. , Basile , G. A. , Buonasera , P. , Pilone , G. , Celeste , G. , Rizzo , G. , Bruschetta , D. , & Cacciola , A. ( 2019 ). The cortico-basal ganglia-cerebellar network: Past, present and future perspectives . Frontiers in Systems Neuroscience , 13 , 61 . 10.3389/fnsys.2019.00061 31736719 PMC6831548

[b59] Morton , S. M. , & Bastian , A. J. ( 2006 ). Cerebellar contributions to locomotor adaptations during splitbelt treadmill walking . Journal of Neuroscience , 26 , 9107 – 9116 . 10.1523/JNEUROSCI.2622-06.2006 16957067 PMC6674518

[b60] Müller , E. J. , Palesi , F. , Hou , K. Y. , Tan , J. , Close , T. , Gandini Wheeler-Kingschott, C. A. M. , D’Angelo , E. , Calamante , F. , & Shine , J. M. ( 2023 ). Parallel processing relies on a distributed, low-dimensional cortico-cerebellar architecture . Network Neuroscience , 7 ( 2 ), 844 – 863 . 10.1162/netn_a_00308 37397895 PMC10312290

[b61] Munn , B. R. , Müller , E. J. , Wainstein , G. , & Shine , J. M. ( 2021 ). The ascending arousal system shapes neural dynamics to mediate awareness of cognitive states . Nature Communications , 12 , 6016 . 10.1038/s41467-021-26268-x PMC8516926 34650039

[b62] Nambu , A. ( 2008 ). Seven problems on the basal ganglia . Current Opinion in Neurobiology , 18 , 595 – 604 . 10.1016/j.conb.2008.11.001 19081243

[b63] Nashef , A. , Rapp , H. , Nawrot , M. P. , & Prut , Y. ( 2018 ). Area-specific processing of cerebellar-thalamo-cortical information in primates . Biological Cybernetics , 112 , 141 – 152 . 10.1007/s00422-017-0738-6 29094187

[b64] Oberhuber , M. , Jones , ‘Ō. P. , Hope , T. M. H. , Prejawa , S. , Seghier , M. L. , Green , D. W. , & Price , C. J. ( 2013 ). Functionally distinct contributions of the anterior and posterior putamen during sublexical and lexical reading . Frontiers in Human Neuroscience , 7 , 787 . 10.3389/fnhum.2013.00787 24312042 PMC3833116

[b65] Oldfield , R. C. ( 1971 ). The assessment and analysis of handedness: The Edinburgh inventory . Neuropsychologia , 9 , 97 – 113 . 10.1016/0028-3932(71)90067-4 5146491

[b66] Pemberton , J. , Chadderton , P. , & Costa , R. P. ( 2022 ). Cerebellar-driven cortical dynamics enable task acquisition, switching and consolidation . bioRxiv . 10.1101/2022.11.14.516257 PMC11686095 39738061

[b67] Perich , M. G. , Gallego , J. A. , & Miller , L. E. ( 2018 ). A neural population mechanism for rapid learning . Neuron , 100 ( 4 ), 964.e7 – 976.e7 . 10.1016/j.neuron.2018.09.030 30344047 PMC6250582

[b68] Perich , M. G. , & Miller , L. E. ( 2017 ). Altered tuning in primary motor cortex does not account for behavioral adaptation during force field learning . Experimental Brain Research , 235 , 2689 – 2704 . 10.1007/s00221-017-4997-1 28589233 PMC5709199

[b69] Prut , Y. , Perlmutter , S. I. , & Fetz , E. E. ( 2001 ). Chapter 17 Distributed processing in the motor system: Spinal cord perspective . Progress in Brain Research , 130 , 267 – 278 . 10.1016/S0079-6123(01)30018-3 11480280

[b70] Ramnani , N. ( 2012 ). Frontal lobe and posterior parietal contributions to the cortico-cerebellar system . Cerebellum , 11 , 366 – 383 . 10.1007/s12311-011-0272-3 21671065

[b71] Ramnani , N. ( 2014 ). Automatic and controlled processing in the corticocerebellar system . Progress in Brain Research , 210 , 255 – 285 . 10.1016/B978-0-444-63356-9.00010-8 24916296

[b72] Ramnani , N. , Behrens , T. E. J. , Johansen-Berg , H. , Richter , M. C. , Pinsk , M. A. , Andersson , J. L. R. , Rudebeck , P. , Ciccarelli , O. , Richter , W. , Thompson , A. J. , Gross , C. G. , Robson , M. D. , Kastner , S. , & Matthews , P. M. ( 2006 ). The evolution of prefrontal inputs to the cortico-pontine system: Diffusion imaging evidence from Macaque Monkeys and Humans . Cerebral Cortex , 16 ( 6 ), 811 – 818 . 10.1093/cercor/bhj024 16120793

[b73] Ramnani , N. , & Miall , R. C. ( 2004 ). A system in the human brain for predicting the actions of others . Nature Neuroscience , 7 , 85 – 90 . 10.1038/nn1168 14699420

[b74] Rotondo , A. P. , Raman , D. V. , & O’Leary , T. ( 2022 ). How cerebellar architecture facilitates rapid online learning . bioRxiv . 10.1101/2022.10.20.512268

[b75] Salo , T. , Yarkoni , T. , Nichols , T. , Poline , J.-B. , Bilgel , M. , Bottenhorn , K. , Jarecka , D. , Kent , J. , Kimbler , A. , Nielson , D. , Oudyk , K. , Peraza , J. , Pérez , A. , Reeders , P. , Yanes , J. , & Laird , A. ( 2022 ). NiMARE: Neuroimaging meta-analysis research environment . NeuroLibre , 1 , 7 . 10.55458/neurolibre.00007

[b76] Santos , S. , Coutinho , D. , Gonçalves , B. , Schöllhorn , W. , Sampaio , J. , & Leite , N. ( 2018 ). Differential learning as a key training approach to improve creative and tactical behavior in soccer . Research Quarterly for Exercise and Sport , 89 , 11 – 24 . 10.1080/02701367.2017.1412063 29351500

[b77] Schaefer , A. , Kong , R. , Gordon , E. M. , Laumann , T. O. , Zuo , X.-N. , Holmes , A. J. , Eickhoff , S. B. , & Yeo , B. T. T. ( 2018 ). Local-global parcellation of the human cerebral cortex from intrinsic functional connectivity MRI . Cerebral Cortex , 28 , 3095 – 3114 . 10.1093/cercor/bhx179 28981612 PMC6095216

[b78] Schmahmann , J. D. , & Pandya , D. N. ( 1997 ). Anatomic organization of the basilar pontine projections from prefrontal cortices in Rhesus Monkey . Journal of Neuroscience , 17 , 438 – 458 . 10.1523/JNEUROSCI.17-01-00438.1997 8987769 PMC6793685

[b79] Schollhorn , W. I. , Hegen , P. , & Davids , K. ( 2012 ). The nonlinear nature of learning—A differential learning approach . The Open Sports Sciences Journal , 5 , 100 – 112 . 10.2174/1875399X01205010100

[b80] Scholz , V. H. , Flaherty , A. W. , Kraft , E. , Keltner , J. R. , Kwong , K. K. , Chen , Y. I. , Rosen , B. R. , & Jenkins , B. G. ( 2000 ). Laterality, somatotopy and reproducibility of the basal ganglia and motor cortex during motor tasks . Brain Research , 879 ( 1–2 ), 204 – 215 . 10.1016/s0006-8993(00)02749-9 11011024

[b81] Sendhilnathan , N. , Bostan , A. C. , Strick , P. L. , & Goldberg , M. E. ( 2024 ). A cerebro-cerebellar network for learning visuomotor associations . Nature Communications , 15 , 2519 . 10.1038/s41467-024-46281-0 PMC10957870 38514616

[b82] Shine , J. M. ( 2021 ). The thalamus integrates the macrosystems of the brain to facilitate complex, adaptive brain network dynamics . Progress in Neurobiology , 199 , 101951 . 10.1016/j.pneurobio.2020.101951 33189781

[b83] Shine , J. M. ( 2022 ). Adaptively navigating affordance landscapes: How interactions between the superior colliculus and thalamus coordinate complex, adaptive behaviour . Neuroscience & Biobehavioral Reviews , 143 , 104921 . 10.1016/j.neubiorev.2022.104921 36280183

[b84] Standage , D. I. , Areshenkoff , C. N. , Gale , D. J. , Nashed , J. Y. , Flanagan , J. R. , & Gallivan , J. P. ( 2023 ). Whole-brain dynamics of human sensorimotor adaptation . Cerebral Cortex , 33 , 4761 – 4778 . 10.1093/cercor/bhac378 36245212 PMC10110437

[b85] Stein , H. ( 2021 ). Why does the neocortex need the cerebellum for working memory ? Journal of Neuroscience , 41 , 6368 – 6370 . 10.1523/JNEUROSCI.0701-21.2021 34321336 PMC8318082

[b86] Striemer , C. L. , Enns , J. T. , & Whitwell , R. L. ( 2019 ). Visuomotor adaptation in the absence of input from early visual cortex . Cortex , 115 , 201 – 215 . 10.1016/j.cortex.2019.01.022 30849551

[b87] Taylor , J. A. , & Ivry , R. B. ( 2011 ). Flexible cognitive strategies during motor learning . PLoS Computational Biology , 7 , e1001096 . 10.1371/journal.pcbi.1001096 21390266 PMC3048379

[b88] Teich , A. F. , & Qian , N. ( 2003 ). Learning and adaptation in a recurrent model of V1 orientation selectivity . Journal of Neurophysiology , 89 , 2086 – 2100 . 10.1152/jn.00970.2002 12611961

[b89] Tian , Y. , Margulies , D. S. , Breakspear , M. , & Zalesky , A. ( 2020 ). Topographic organization of the human subcortex unveiled with functional connectivity gradients . Nature Neuroscience , 23 , 1421 – 1432 . 10.1038/s41593-020-00711-6 32989295

[b90] Todorov , D. I. , Capps , R. A. , Barnett , W. H. , Latash , E. M. , Kim , T. , Hamade , K. C. , Markin , S. N. , Rybak , I. A. , & Molkov , Y. I. ( 2019 ). The interplay between cerebellum and basal ganglia in motor adaptation: A modeling study . PLoS One , 14 , e0214926 . 10.1371/journal.pone.0214926 30978216 PMC6461234

[b91] Tsay , J. S. , Kim , H. , Haith , A. M. , & Ivry , R. B. ( 2022 ). Understanding implicit sensorimotor adaptation as a process of proprioceptive re-alignment . eLife , 11 , e76639 . 10.7554/eLife.76639 35969491 PMC9377801

[b92] Tseng , Y. , Diedrichsen , J. , Krakauer , J. W. , Shadmehr , R. , & Bastian , A. J. ( 2007 ). Sensory prediction errors drive cerebellum-dependent adaptation of reaching . Journal of Neurophysiology , 98 , 54 – 62 . 10.1152/jn.00266.2007 17507504

[b93] Tzvi , E. , Koeth , F. , Karabanov , A. N. , Siebner , H. R. , & Krämer , U. M. ( 2020 ). Cerebellar—Premotor cortex interactions underlying visuomotor adaptation . NeuroImage , 220 , 117142 . 10.1016/j.neuroimage.2020.117142 32634591

[b94] Tzvi , E. , Loens , S. , & Donchin , O. ( 2022 ). Mini-review: The role of the cerebellum in visuomotor adaptation . Cerebellum , 21 , 306 – 313 . 10.1007/s12311-021-01281-4 34080132 PMC8993777

[b95] Viñas-Guasch , N. , & Wu , Y. J. ( 2017 ). The role of the putamen in language: A meta-analytic connectivity modeling study . Brain Structure and Function , 222 , 3991 – 4004 . 10.1007/s00429-017-1450-y 28585051

[b96] Vinh , N. X. , & Epps , J. ( 2009 ). A novel approach for automatic number of clusters detection in microarray data based on consensus clustering . In 2009 Ninth IEEE International Conference on Bioinformatics and BioEngineering, Taichung, Taiwan (pp. 84 – 91 ). IEEE . 10.1109/BIBE.2009.19

[b97] Vinh , N.X. , Epps , J. , Bailey , J . ( 2009 ). Information theoretic measures for clusterings comparison: Is a correction for chance necessary? In Proceedings of the 26th Annual International Conference on Machine Learning , 1073 – 1080 . 10.1145/1553374.1553511

[b98] Vinh , N. X. , Epps , J. , & Bailey , J. ( 2010 ). Information theoretic measures for clusterings comparison: variants, properties, normalization and correction for chance . Journal of Machine Learning Research , 11 , 2837 – 2854 .

[b99] Wagner , M. J. , Kim , T. H. , Kadmon , J. , Nguyen , N. D. , Ganguli , S. , Schnitzer , M. J. , & Luo , L. ( 2019 ). Shared cortex-cerebellum dynamics in the execution and learning of a motor task . Cell , 177 , 669.e24 – 682.e24 . 10.1016/j.cell.2019.02.019 30929904 PMC6500577

[b100] Wilson , C. J. ( 2013 ). Active decorrelation in the basal ganglia . Neuroscience , 250 , 467 – 482 . 10.1016/j.neuroscience.2013.07.032 23892007 PMC3772785

[b101] Wolff , S. B. E. , Ko , R. , & Ölveczky , B. P. ( 2022 ). Distinct roles for motor cortical and thalamic inputs to striatum during motor skill learning and execution . Science Advances , 8 , eabk0231 . 10.1126/sciadv.abk0231 35213216 PMC8880788

[b102] Wolpert , D. M. , & Flanagan , J. R. ( 2001 ). Motor prediction . Current Biology , 11 , R729 – R732 . 10.1016/S0960-9822(01)00432-8 11566114

[b103] Wolpert , D. M. , Miall , R. C. , & Kawato , M. ( 1998 ). Internal models in the cerebellum . Trends in Cognitive Sciences , 2 , 338 – 347 . 10.1016/S1364-6613(98)01221-2 21227230

[b200] World Medical Association . ( 2013 ). Declaration of Helsinki: Ethical principles for medical research involving human subjects . JAMA , 310 , 2191 – 2194 . 10.1001/jama.2013.281053 24141714

[b104] Xue , A. , Kong , R. , Yang , Q. , Eldaief , M. C. , Angeli , P. A. , DiNicola , L. M. , Braga , R. M. , Buckner , R. L. , & Yeo , B. T. T. ( 2021 ). The detailed organization of the human cerebellum estimated by intrinsic functional connectivity within the individual . Journal of Neurophysiology , 125 , 358 – 384 . 10.1152/jn.00561.2020 33427596 PMC7948146

[b105] Yang , J. ( 2015 ). The influence of motor expertise on the brain activity of motor task performance: A meta-analysis of functional magnetic resonance imaging studies . Cognitive, Affective, & Behavioral Neuroscience , 15 , 381 – 394 . 10.3758/s13415-014-0329-0 25450866

[b106] Yarkoni , T. , Poldrack , R. A. , Nichols , T. E. , Van Essen , D. C. , & Wager , T. D. ( 2011 ). Large-scale automated synthesis of human functional neuroimaging data . Nature Methods , 8 , 665 – 670 . 10.1038/nmeth.1635 21706013 PMC3146590

[b107] Yin , H. H. , & Knowlton , B. J. ( 2006 ). The role of the basal ganglia in habit formation . Nature Reviews Neuroscience , 7 , 464 – 476 . 10.1038/nrn1919 16715055

[b108] Zagon , I. S. , McLaughlin , P. J. , & Smith , S. ( 1977 ). Neural populations in the human cerebellum: Estimations from isolated cell nuclei . Brain Research , 127 , 279 – 282 . 10.1016/0006-8993(77)90541-8 861760

[b109] Zinger , N. , Harel , R. , Gabler , S. , Israel , Z. , & Prut , Y. ( 2013 ). Functional organization of information flow in the corticospinal pathway . Journal of Neuroscience , 33 , 1190 – 1197 . 10.1523/JNEUROSCI.2403-12.2013 23325255 PMC6704852

