# Supplementary material for: The engagement of the cerebellum and basal ganglia enhances expertise in a sensorimotor adaptation task
Source: Imaging Neurosci (Camb). 2024 Aug 19;2:imag-2-00271. doi: 10.1162/imag_a_00271 (PMC12290584; doi:10.1162/imag_a_00271)
Supplement: Supplementary Material [file imag_a_00271-supp.pdf]

Supplementary Material

Cerebellar engagement enhances expertise in a sensorimotor adaptation task

1. fMRI preprocessing Page 2

2. fMRI scan timing Page 5

3. Table of correlation coefficients for significant regions in BIS GLM Page 6

4. Schaefer 1000 analysis Page 10

1. fMRI preprocessing

Anatomical data preprocessing

A total of 2 T1-weighted (T1w) images were found within the input BIDS dataset. All of them were corrected for intensity non-uniformity (INU) with `N4BiasFieldCorrection` [@n4], distributed with ANTs 2.3.1 [@ants, RRID:SCR_004757]. The T1w-reference was then skull-stripped with a *Nipype* implementation of the `antsBrainExtraction.sh` workflow (from ANTs), using OASIS30ANTs as target template. Brain tissue segmentation of cerebrospinal fluid (CSF), white-matter (WM) and gray-matter (GM) was performed on the brain-extracted T1w using `fast` [FSL 6.0.3:b862cdd5, RRID:SCR_002823, @fsl_fast]. A T1w-reference map was computed after registration of 2 T1w images (after INU-correction) using `mri_robust_template` [FreeSurfer 7.1.1, @fs_template]. Volume-based spatial normalization to one standard space (MNI152NLin2009cAsym) was performed through nonlinear registration with `antsRegistration` (ANTs 2.3.1), using brain-extracted versions of both T1w reference and the T1w template. The following template was selected for spatial normalization: *ICBM 152 Nonlinear Asymmetrical template version 2009c* [@mni152nlin2009casym, RRID:SCR_008796; TemplateFlow ID: NI152NLin2009cAsym].

Functional data preprocessing

For each of the 2 BOLD runs found per subject (across all tasks and sessions), the following preprocessing was performed. First, a reference volume and its skull-stripped version were generated using a custom methodology of *fMRIPrep*. Head-motion parameters with respect to the BOLD reference (transformation matrices, and six corresponding rotation and translation parameters) are estimated before any spatiotemporal filtering using `mcflirt` [FSL 6.0.3:b862cdd5, @mcflirt]. BOLD runs were slice-time corrected to 0.975s (0.5 of slice acquisition range 0s-1.95s) using `3dTshift` from AFNI 20170202 [@afni, RRID:SCR_005927].

The BOLD time-series (including slice-timing correction when applied) were resampled onto their original, native space by applying the transforms to correct for head-motion. These resampled BOLD time-series will be referred to as *preprocessed BOLD in original space*, or just *preprocessed BOLD*. The BOLD reference was then co-registered to the T1w reference using `mri_coreg` (FreeSurfer) followed by `flirt`

[FSL 6.0.3:b862cdd5, @flirt] with the boundary-based registration [@bbr] cost-function. Co-registration was configured with six degrees of freedom.
Several confounding time-series were calculated based on the *preprocessed BOLD*: framewise displacement (FD), DVARS and three region-wise global signals. FD was computed using two formulations following Power (absolute sum of relative motions, @power_fd_dvars) and Jenkinson (relative root mean square displacement between affines, @mcflirt). FD and DVARS are calculated for each functional run, both using their implementations in *Nipype* [following the definitions by @power_fd_dvars]. The three global signals are extracted within the CSF, the WM, and the whole-brain masks. Additionally, a set of physiological regressors were extracted to allow for component-based noise correction [*CompCor*, @compcor]. Principal components are estimated after high-pass filtering the *preprocessed BOLD* time-series (using a discrete cosine filter with 128s cut-off) for the two *CompCor* variants: temporal (tCompCor) and anatomical (aCompCor). tCompCor components are then calculated from the top 2% variable voxels within the brain mask. For aCompCor, three probabilistic masks (CSF, WM and combined CSF+WM) are generated in anatomical space. The implementation differs from that of Behzadi et al. in that instead of eroding the masks by 2 pixels on BOLD space, the aCompCor masks are subtracted a mask of pixels that likely contain a volume fraction of GM. This mask is obtained by thresholding the corresponding partial volume map at 0.05, and it ensures components are not extracted from voxels containing a minimal fraction of GM. Finally, these masks are resampled into BOLD space and binarized by thresholding at 0.99 (as in the original implementation). Components are also calculated separately within the WM and CSF masks. For each CompCor decomposition, the *k* components with the largest singular values are retained, such that the retained components' time series are sufficient to explain 50 percent of variance across the nuisance mask (CSF, WM, combined, or temporal). The remaining components are dropped from consideration. The head-motion estimates calculated in the correction step were also placed within the corresponding confounds file. The confound time series derived from head motion estimates and global signals were expanded with the inclusion of temporal derivatives and quadratic terms for each [@confounds_satterthwaite_2013]. Frames that exceeded a threshold of 0.5 mm FD or 1.5 standardised DVARS were annotated as motion outliers.

The BOLD time-series were resampled into standard space, generating a *preprocessed BOLD run in MNI152NLin2009cAsym space*. First, a reference volume and its skull-stripped version were generated using a custom methodology of *fMRIPrep*. All resamplings can be performed with *a single interpolation step* by composing all the pertinent transformations (i.e. head-motion transform matrices, susceptibility distortion correction when available, and co-registrations to anatomical and output spaces). Gridded (volumetric) resamplings were performed using `antsApplyTransforms` (ANTs), configured with Lanczos interpolation to minimize the smoothing effects of other kernels [@lanczos]. Non-gridded (surface) resamplings were performed using `mri_vol2surf` (FreeSurfer).

Many internal operations of *fMRIPrep* use *Nilearn* 0.9.1 [@nilearn, RID:SCR_001362], mostly within the functional processing workflow. For more details of the pipeline, see [the section corresponding to workflows in *fMRIPrep*'s documentation] (https://fmriprep.readthedocs.io/en/latest/workflows.html "FMRIPrep's documentation").

2. fMRI scan timing

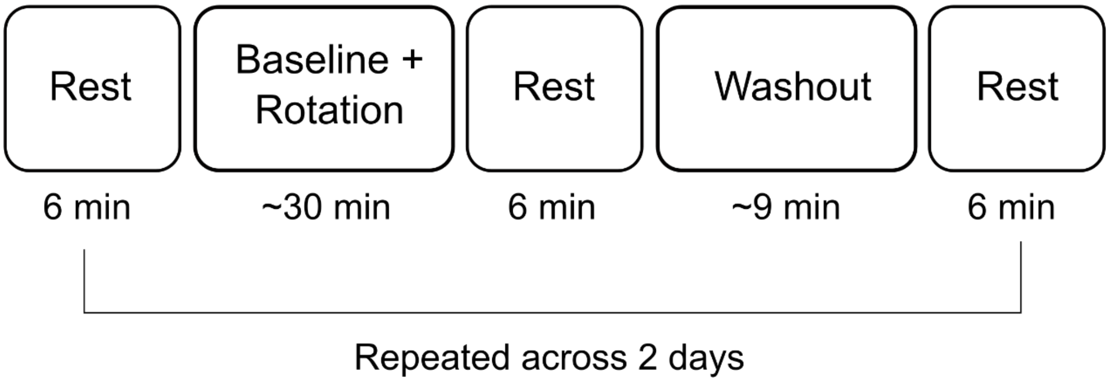

Figure 7 Breakdown of fMRI scan timing. fMRI scanning was spread across two days. Each day participants underwent three 6 min resting state scans prior to, in-between, and after the task scans. The first task scan was ~30 min consisting of the Baseline and Rotation conditions. The second task scan was ~9 min and consisted of the Washout condition. The total scanning time each day was 75 min including setup time.

3. Table of correlation coefficients for significant regions in BIS GLM

ROI Name	MNI Coordinates			GLM Coefficient
	X	Y	Z	
17Networks_LH_VisCent_ExStr_5	-24	-97	-12	0.05462
17Networks_LH_VisPeri_ExStrInf_5	-14	-57	1	-0.0255
17Networks_LH_VisPeri_ExStrSup_1	-19	-65	7	-0.0265
17Networks_LH_SomMotA_1	-8	-15	47	-0.0639
17Networks_LH_SomMotA_3	-49	-17	54	-0.0913
17Networks_LH_SomMotA_4	-48	-29	58	-0.1062
17Networks_LH_SomMotA_5	-39	-25	53	-0.1118
17Networks_LH_SomMotA_8	-4	-9	59	-0.0552
17Networks_LH_SomMotA_9	-36	-19	65	-0.1091
17Networks_LH_SomMotA_10	-32	-29	63	-0.1062
17Networks_LH_SomMotA_11	-30	-38	65	-0.064
17Networks_LH_SomMotA_12	-23	-11	65	-0.0819
17Networks_LH_SomMotA_13	-19	-24	67	-0.0404
17Networks_LH_SomMotA_16	-14	-11	73	-0.0498
17Networks_LH_SomMotA_19	-12	-27	73	-0.0348
17Networks_LH_SomMotB_Ins_1	-36	-24	10	-0.0271
17Networks_LH_SomMotB_S2_2	-36	-26	19	-0.0436
17Networks_LH_SomMotB_S2_3	-49	-13	14	-0.0258
17Networks_LH_SomMotB_S2_5	-48	-24	18	-0.06
17Networks_LH_DorsAttnA_ParOcc_1	-48	-65	15	-0.0331
17Networks_LH_DorsAttnA_SPL_3	-23	-65	46	-0.029
17Networks_LH_DorsAttnA_SPL_4	-29	-58	50	-0.0255
17Networks_LH_DorsAttnA_SPL_5	-36	-52	56	-0.0364
17Networks_LH_DorsAttnA_SPL_6	-15	-71	57	-0.0363
17Networks_LH_DorsAttnA_SPL_7	-29	-61	62	-0.0409
17Networks_LH_DorsAttnB_PostC_1	-61	-23	33	-0.0347
17Networks_LH_DorsAttnB_PostC_2	-55	-20	41	-0.0824
17Networks_LH_DorsAttnB_PostC_3	-55	-32	45	-0.0433
17Networks_LH_DorsAttnB_PostC_4	-46	-29	44	-0.0732
17Networks_LH_DorsAttnB_PostC_5	-39	-37	49	-0.0921
17Networks_LH_DorsAttnB_PostC_6	-30	-46	63	-0.0725
17Networks_LH_DorsAttnB_PostC_7	-7	-59	63	-0.0546
17Networks_LH_DorsAttnB_PostC_8	-20	-57	66	-0.0508
17Networks_LH_DorsAttnB_PostC_9	-13	-50	72	-0.0238
17Networks_LH_DorsAttnB_FEF_2	-25	-1	55	-0.0617
17Networks_LH_DorsAttnB_FEF_3	-30	-8	52	-0.0797
17Networks_LH_DorsAttnB_PrCv_1	-50	3	38	-0.0346

17Networks_LH_SalVentAttnA_ParOper_1	-55	-32	22	-0.0272
17Networks_LH_SalVentAttnA_ParOper_3	-61	-36	33	-0.0369
17Networks_LH_SalVentAttnA_Ins_1	-39	2	-4	-0.0268
17Networks_LH_SalVentAttnA_Ins_2	-40	-15	-2	-0.0287
17Networks_LH_SalVentAttnA_Ins_3	-33	19	8	-0.0537
17Networks_LH_SalVentAttnA_FrOper_2	-52	9	13	-0.0282
17Networks_LH_SalVentAttnA_ParMed_3	-6	-49	57	-0.0437
17Networks_LH_SalVentAttnA_FrMed_1	-7	0	41	-0.0404
17Networks_LH_SalVentAttnA_FrMed_2	-5	9	48	-0.0649
17Networks_LH_SalVentAttnB_PFCl_2	-29	43	30	-0.0292
17Networks_LH_SalVentAttnB_PFCl_3	-36	32	38	-0.0321
17Networks_LH_SalVentAttnB_Ins_2	-33	25	-1	-0.0593
17Networks_LH_SalVentAttnB_Ins_3	-43	12	2	-0.0328
17Networks_LH_SalVentAttnB_PFCmp_1	-6	22	31	-0.03
17Networks_LH_LimbicA_TempPole_4	-54	-21	-31	-0.0266
17Networks_LH_LimbicA_TempPole_6	-32	12	-29	-0.0276
17Networks_LH_LimbicA_TempPole_7	-44	5	-17	-0.0279
17Networks_LH_ContA_IPS_4	-45	-41	47	-0.0537
17Networks_LH_ContA_IPS_5	-33	-46	41	-0.045
17Networks_LH_ContA_PFCd_1	-21	5	65	-0.0515
17Networks_LH_ContA_PFClv_2	-42	38	22	-0.0256
17Networks_LH_ContA_PFCl_1	-49	6	26	-0.0325
17Networks_LH_ContA_PFCl_2	-45	20	27	-0.0295
17Networks_LH_ContA_PFCl_3	-39	7	34	-0.0467
17Networks_LH_ContB_IPL_3	-42	-52	49	-0.0378
17Networks_LH_ContB_PFCd_1	-30	14	57	-0.0232
17Networks_LH_ContB_PFCmp_1	-4	28	47	-0.0508
17Networks_LH_ContC_pCun_3	-5	-64	52	-0.044
17Networks_LH_DefaultA_PFCd_2	-18	36	48	0.03881
17Networks_LH_DefaultA_pCunPCC_3	-7	-44	32	0.02499
17Networks_LH_DefaultA_pCunPCC_5	-3	-15	37	0.03063
17Networks_LH_DefaultA_PFCm_3	-6	59	7	0.03513
17Networks_LH_DefaultB_Temp_2	-54	-2	-30	-0.0247
17Networks_LH_DefaultB_PFCd_6	-6	10	65	-0.0281
17Networks_LH_DefaultB_PFCv_1	-36	22	-16	-0.0269
17Networks_LH_DefaultC_IPL_1	-40	-79	30	-0.0304
17Networks_RH_VisCent_ExStr_5	42	-84	-12	0.03368
17Networks_RH_VisCent_ExStr_6	25	-97	-10	0.06323
17Networks_RH_VisCent_ExStr_7	35	-89	2	0.07488
17Networks_RH_VisCent_ExStr_8	24	-99	7	0.04861
17Networks_RH_VisPeri_ExStrInf_4	13	-58	-3	-0.0256
17Networks_RH_VisPeri_ExStrInf_5	18	-45	-3	-0.0315

17Networks_RH_VisPeri_StriCal_2	22	-59	6	-0.0341
17Networks_RH_VisPeri_ExStrSup_4	16	-87	36	0.03028
17Networks_RH_SomMotA_1	54	-17	40	-0.0808
17Networks_RH_SomMotA_4	49	-26	56	-0.0303
17Networks_RH_SomMotA_5	7	-10	51	-0.0327
17Networks_RH_SomMotA_7	37	-20	64	-0.0329
17Networks_RH_SomMotA_12	29	-11	65	-0.077
17Networks_RH_SomMotA_17	17	-6	69	-0.0345
17Networks_RH_SomMotA_19	17	-18	73	-0.0317
17Networks_RH_SomMotB_S2_7	49	-21	19	-0.0301
17Networks_RH_SomMotB_Cent_1	61	6	30	-0.0334
17Networks_RH_DorsAttnA_SPL_5	21	-69	53	-0.0338
17Networks_RH_DorsAttnA_SPL_6	34	-50	54	-0.0367
17Networks_RH_DorsAttnA_SPL_7	27	-58	61	-0.0313
17Networks_RH_DorsAttnA_SPL_8	14	-64	65	-0.0366
17Networks_RH_DorsAttnB_PostC_1	61	-14	30	-0.0476
17Networks_RH_DorsAttnB_PostC_2	57	-23	44	-0.0446
17Networks_RH_DorsAttnB_PostC_3	44	-37	50	-0.0529
17Networks_RH_DorsAttnB_PostC_4	45	-28	42	-0.0575
17Networks_RH_DorsAttnB_PostC_5	35	-36	51	-0.0624
17Networks_RH_DorsAttnB_PostC_6	7	-54	59	-0.0502
17Networks_RH_DorsAttnB_PostC_7	24	-50	68	-0.0421
17Networks_RH_DorsAttnB_PostC_8	16	-47	74	-0.0241
17Networks_RH_DorsAttnB_FEF_2	27	-3	52	-0.0692
17Networks_RH_DorsAttnB_FEF_3	25	-3	64	-0.056
17Networks_RH_SalVentAttnA_ParOper_3	63	-26	38	-0.0293
17Networks_RH_SalVentAttnA_FrOper_3	54	12	12	-0.0295
17Networks_RH_SalVentAttnA_FrMed_1	7	2	43	-0.0434
17Networks_RH_SalVentAttnA_FrMed_2	6	11	58	-0.0678
17Networks_RH_SalVentAttnA_ParMed_3	10	-43	53	-0.0335
17Networks_RH_SalVentAttnA_FrMed_3	7	-2	67	-0.0344
17Networks_RH_SalVentAttnA_FrMed_4	16	7	69	-0.0548
17Networks_RH_SalVentAttnB_IPL_1	62	-37	37	-0.0313
17Networks_RH_SalVentAttnB_PFCl_2	25	54	25	-0.0278
17Networks_RH_SalVentAttnB_PFCl_3	33	45	28	-0.0388
17Networks_RH_SalVentAttnB_Ins_2	37	23	5	-0.0445
17Networks_RH_SalVentAttnB_PFCmp_1	8	35	25	-0.0248
17Networks_RH_SalVentAttnB_PFCmp_2	7	19	35	-0.0555
17Networks_RH_LimbicB_OFC_2	23	22	-21	-0.0199
17Networks_RH_ContA_IPS_2	54	-33	51	-0.0342
17Networks_RH_ContA_IPS_3	47	-44	46	-0.0332
17Networks_RH_ContA_IPS_4	36	-44	45	-0.0426

17Networks_RH_ContA_PFCd_1	24	10	58	-0.0545
17Networks_RH_ContA_PFCl_3	47	29	28	-0.035
17Networks_RH_ContA_PFCl_4	49	8	25	-0.038
17Networks_RH_ContA_PFCl_5	39	11	34	-0.0274
17Networks_RH_ContB_PFCld_1	39	33	38	-0.0337
17Networks_RH_ContB_PFCld_2	45	19	44	-0.0322
17Networks_RH_ContB_PFCld_3	43	7	51	-0.0218
17Networks_RH_ContB_PFCld_4	34	15	56	-0.029
17Networks_RH_ContB_PFCmp_1	5	28	48	-0.0533
17Networks_RH_ContC_pCun_1	17	-63	28	-0.0296
17Networks_RH_ContC_pCun_3	5	-64	44	-0.0282
17Networks_RH_ContC_pCun_5	8	-71	53	-0.037
17Networks_RH_DefaultA_pCunPCC_2	5	-63	31	0.02473
17Networks_RH_DefaultA_pCunPCC_5	10	-53	35	0.01765
17Networks_RH_DefaultA_PFCm_3	7	42	4	0.02618
17Networks_RH_DefaultB_PFCv_2	48	32	-8	-0.0277
17Networks_RH_DefaultC_IPL_1	48	-64	22	-0.0306
17Networks_RH_DefaultC_IPL_2	45	-75	31	-0.0433
Right_V	18	-50	-19	-0.0476
Right_CrusI	-45	-65	-32	0.0254
Right_IX	5	-51	-55	-0.0279
Right_X	22	-35	-43	-0.0215
PUT-VA-rh	22	12	-6	0.05165
PUT-DA-rh	26	6	2	0.03852
PUT-VP-rh	30	-12	0	0.03365
PUT-DP-rh	28	-2	6	0.0307
PUT-DA-lh	-24	6	2	0.03217
CAU-tail-lh	-14	-2	20	-0.0263
aGP-lh	-16	0	-2	-0.0221

4. Schaefer 1000 analysis

The general linear model results conducted with the Voltron-400 atlas was replicated with using the Voltron-1000 atlas. The main difference between the two atlas' is the use of 1000 Schaefer cortical nodes, instead of 400 Schaefer cortical nodes. The rest of the regions of the interests (basal ganglia, cerebellum) remain the same.

Improved performance is driven by basal ganglia and cerebellar engagement

The peak BOLD response for each region across all trials was fit to BIS in a general linear model. A one-sample test was used to create a thresholded group map (Figure 7A, $p < 0.05$). Regions that increased in BOLD with better performance included the right Crus I of the cerebellum, right putamen, and sparse activation across the medial cerebral cortex including regions such as the medial prefrontal cortex and cingulate cortex. Regions that were associated with worse performance included bilaterally the primary motor, parietal, extrastriate cortices, as well as the right frontal lobe.

Balanced Integration Score (BIS) retains insights from Response Time (RT) and Response Error (RE) in granular parcellations

The BIS group map was compared to group maps fitted with response time (RT) and response error (RE). From Pearson's correlations, the BIS map was significantly similar to both the RT and RE brain maps (Figure 7B, 7C; $r = 0.75$, $p = 0$; $r = 0.73$, $p = 0$, respectively). To compare whether there were differences between task conditions, the BIS scores and BOLD responses were divided into four conditions (baseline, early rotation, late rotation, and washout). All conditions were similar to each other, and the strongest correlation was between the baseline and late rotation (Figure 7D; $r = 0.63$, $p = 0$).

Expertise brain maps were robust to individual effects

After dividing the dataset in half and fitting each group to their BIS scores in two separate GLMs, the average beta coefficients were compared between the two groups. The beta coefficients between the two groups were strongly correlated (Figure 7E; $r = 0.62$, $p = 0$), providing evidence that the current results were not driven by individual subjects.

All cerebral cortical correlations were corrected for spatial autocorrelation using spin-permutation tests, and all comparisons remained significantly different ($p_{spin} = 0$).

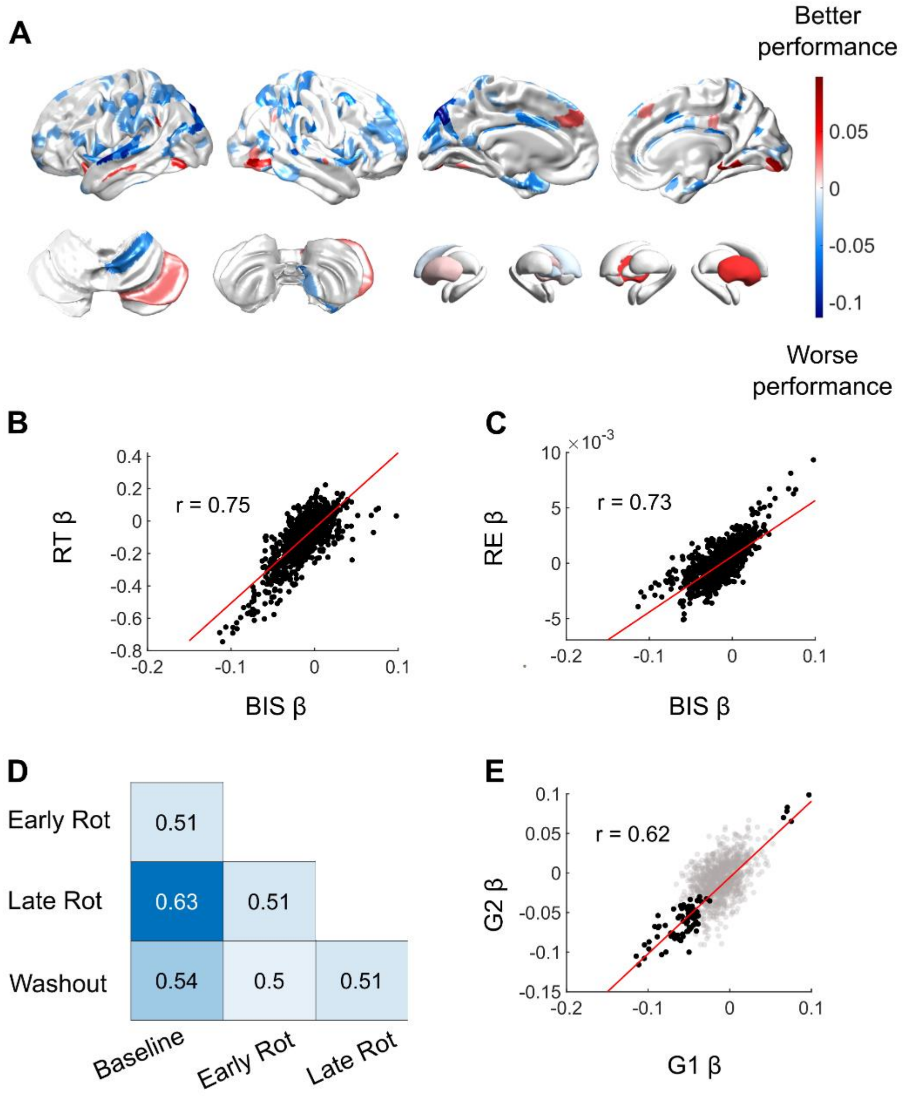

Figure 8 Summary of general linear modelling in the Voltron-1000 parcellation. A. Thresholded brain maps of beta coefficients comparing BIS against peak BOLD response across all trials. B. Comparison of BIS beta coefficients against response time (RT) beta coefficients. C. Comparison of BIS beta coefficients against response error (RE) beta coefficients. D. Comparison of beta coefficients for each condition. E. The dataset was divided into two groups (n = 12, n = 11). Comparison of group 1 BIS beta coefficients against group 2 BIS beta coefficients.

References

Abraham, Alexandre, *et al.* (2014) "Machine Learning for Neuroimaging with Scikit-Learn." Frontiers in Neuroinformatics 8. https://doi.org/10.3389/fninf.2014.00014.

Avants, B.B., C.L. Epstein, M. Grossman, and J.C. Gee (2008) "Symmetric Diffeomorphic Image Registration with Cross-Correlation: Evaluating Automated Labeling of Elderly and Neurodegenerative Brain." Medical Image Analysis 12 (1): 26–41. https://doi.org/10.1016/j.media.2007.06.004.

Behzadi, Yashar, Khaled Restom, Joy Liau, and Thomas T. Liu. (2007) "A Component Based Noise Correction Method (CompCor) for BOLD and Perfusion Based fMRI." *NeuroImage* 37 (1): 90–101. https://doi.org/10.1016/j.neuroimage.2007.04.042.

Esteban, Oscar, *et al.* (2018) "FMRIPrep." *Software*. Zenodo. https://doi.org/10.5281/zenodo.852659.

Esteban, Oscar, *et al.* (2018) "fMRIPrep: A Robust Preprocessing Pipeline for Functional MRI." *Nature Methods*. https://doi.org/10.1038/s41592-018-0235-4.

Evans, AC, *et al.* (2012) "Brain Templates and Atlases." *NeuroImage* 62 (2): 911–22. https://doi.org/10.1016/j.neuroimage.2012.01.024.

Fonov, VS, *et al.* (2009) "Unbiased Nonlinear Average Age-Appropriate Brain Templates from Birth to Adulthood." *NeuroImage* 47, Supplement 1: S102. https://doi.org/10.1016/S1053-8119(09)70884-5.

Gorgolewski, K., *et al.* (2011) "Nipype: A Flexible, Lightweight and Extensible Neuroimaging Data Processing Framework in Python." *Frontiers in Neuroinformatics* 5: 13. https://doi.org/10.3389/fninf.2011.00013.

Gorgolewski, Krzysztof J. *et al*. (2018) "Nipype." *Software*. Zenodo. https://doi.org/10.5281/zenodo.596855.

Greve, Douglas N, and Bruce Fischl (2009) "Accurate and Robust Brain Image Alignment Using Boundary-Based Registration." *NeuroImage* 48 (1): 63–72. https://doi.org/10.1016/j.neuroimage.2009.06.060.

Jenkinson, Mark, *et al.* (2002) “Improved Optimization for the Robust and Accurate Linear Registration and Motion Correction of Brain Images.” *NeuroImage* 17 (2): 825–41. https://doi.org/10.1006/nimg.2002.1132.

Jenkinson, Mark, and Stephen Smith (2001) “A Global Optimisation Method for Robust Affine Registration of Brain Images.” *Medical Image Analysis* 5 (2): 143–56. https://doi.org/10.1016/S1361-8415(01)00036-6.

Lanczos, C. (1964) “Evaluation of Noisy Data.” *Journal of the Society for Industrial and Applied Mathematics Series B Numerical Analysis* 1 (1): 76–85. https://doi.org/10.1137/0701007.

Power, Jonathan D. *et al.* (2014) “Methods to Detect, Characterize, and Remove Motion Artifact in Resting State fMRI.” *NeuroImage* 84 (Supplement C): 320–41. https://doi.org/10.1016/j.neuroimage.2013.08.048.

Pruim, Raimon H. R. *et al.* (2015) “ICA-AROMA: A Robust ICA-Based Strategy for Removing Motion Artifacts from fMRI Data.” *NeuroImage* 112 (Supplement C): 267–77. https://doi.org/10.1016/j.neuroimage.2015.02.064.

Satterthwaite, T.D. *et al.* (2013) ‘An improved framework for confound regression and filtering for control of motion artifact in the preprocessing of resting-state functional connectivity data’, *NeuroImage*, 64, pp. 240–256. Available at: https://doi.org/10.1016/j.neuroimage.2012.08.052.

Thomas Yeo, B.T. *et al.* (2011) ‘The organization of the human cerebral cortex estimated by intrinsic functional connectivity’, *Journal of Neurophysiology*, 106(3), pp. 1125–1165. Available at: https://doi.org/10.1152/jn.00338.2011.

Tustison, N. J. *et al.* (2010) “N4ITK: Improved N3 Bias Correction.” *IEEE Transactions on Medical Imaging* 29 (6): 1310–20. https://doi.org/10.1109/TMI.2010.2046908.

Zhang, Y., M. Brady, and S. Smith, (2001) “Segmentation of Brain MR Images Through a Hidden Markov Random Field Model and the Expectation-Maximization Algorithm.” *IEEE Transactions on Medical Imaging* 20 (1): 45–57. https://doi.org/10.1109/42.906424.
